# AIMNet2‐NSE: A Transferable Reactive Neural Network Potential for Open‐Shell Chemistry

**DOI:** 10.1002/anie.202516763

**Published:** 2025-12-16

**Authors:** Bhupalee Kalita, Roman Zubatyuk, Dylan M. Anstine, Maike Bergeler, Volker Settels, Conrad Stork, Sebastian Spicher, Olexandr Isayev

**Affiliations:** ^1^ Department of Chemistry Carnegie Mellon University Pittsburgh PA 15213 United States; ^2^ Department of Chemical Engineering and Materials Science Michigan State University East Lansing MI 48824 United States; ^3^ BASF SE Carl‐Bosch Straße 38 Ludwigshafen am Rhein 67056 Germany; ^4^ Department of Materials Science and Engineering Carnegie Mellon University Pittsburgh PA 15213 United States

**Keywords:** Computational chemistry, Machine learning interatomic potential, Open‐shell chemistry, Polymerization, Radical reactions

## Abstract

Open‐shell systems such as radical intermediates are central to radical polymerization (RP), combustion, catalysis, and many other chemical and industrial processes, yet their accurate modeling presents significant computational challenges. Most of the current machine learning interatomic potentials do not distinguish between different spin states, making them unsuitable for open‐shell reactive chemistry. Here we present AIMNet2‐NSE (neural spin‐charge equilibration), a neural network potential that incorporates spin‐charge equilibration for accurate treatment of molecules and reactions with arbitrary charge and spin multiplicities. Built upon the AIMNet2 framework, AIMNet2‐NSE is trained on an extensive dataset comprising 20 million closed‐shell neutral and charged molecules, 13 million open‐shell radical configurations, and 200K radical reaction profiles. With explicit handling of spin charges, AIMNet2‐NSE enables prediction of spin‐resolved properties with near‐DFT accuracy while maintaining a favorable linear scaling compared to the polynomial scaling of electronic structure methods. The predictive capabilities and generalizability of our model are confirmed by evaluations on large‐scale radical test sets, the industrially relevant BASChem19 benchmark, and RP reactions. Overall, AIMNet2‐NSE represents a significant advancement in machine learning interatomic potentials, allowing efficient exploration of complex open‐shell systems, and significantly advancing our ability to model radical reaction pathways and reactive intermediates in chemical processes where traditional quantum mechanical methods are computationally prohibitive.

## Graphical abstract

AIMNet2‐NSE enables accurate modeling of open‐shell radical chemistry through neural spin‐charge equilibration (NSE). This machine learning interatomic potential predicts radical reaction energies, barriers, and spin‐crossing events with near‐DFT accuracy while offering five orders of magnitude computational speedup, making high‐throughput exploration of radical intermediates feasible for industrial applications.

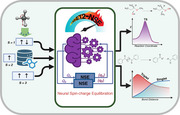

## Introduction

Open‐shell systems, characterized by the presence of unpaired electrons, are critical for a variety of chemical and biological processes, including radical‐mediated reactions,^[^ ^1^ ^]^ combustion chemistry,^[^ ^2^ ^]^ enzymatic transformations,^[^ ^3^ ^]^ plasmas,^[^ ^4^ ^]^ atmospheric chemistry,^[^ ^5^ ^]^ and interstellar environments.^[^ ^6^ ^]^ Understanding the chemical reactivity of open‐shell species is pivotal to assess, and ultimately tailor, several reaction mechanisms. Radical polymerization (RP) reactions, for example, are responsible for approximately 45% of all industrially manufactured polymers.^[^ ^7^ ^]^ Their versatility and adaptability have made them indispensable for producing high‐volume thermoplastics like polyethylene and polypropylene,^[^ ^8^ ^]^ photo‐polymerized coatings,^[^ ^9^, ^10^ ^]^ adhesives,^[^ ^11^ ^]^ and numerous advanced engineered materials.^[^ ^12^, ^13^ ^]^ These applications span critical sectors, including automotive, construction, electronics, innovations in lightweight materials, and sustainability‐focused solutions.^[^ ^7^, ^14^, ^15^, ^16^, ^17^, ^18^ ^]^

Experimentally, it is challenging to characterize free radicals and their reactions due to the difficulty in their preparation and their highly reactive nature under typical laboratory or industrial conditions.^[^ ^1^, ^19^ ^]^ Alternatively, one can use electronic structure methods with varying degrees of approximations to understand the underlying physics that governs chemical reactivity of radicals. However, accurate treatment of reactivities and energetics of radical species presents significant challenges associated with their open‐shell electronic structure, including potential multi‐reference character and closely lying electronic states. Radical reaction pathways may also involve transition between different spin states, necessitating methods that can handle spin‐crossing events across diverse molecular configurations rather than just near equilibrium structures.^[^ ^20^ ^]^ Methods such as unrestricted density functional theory (DFT)^[^ ^21^, ^22^ ^]^ are commonly preferred for studying open‐shell systems. While DFT can provide a detailed description of potential energy surfaces (PES), it may suffer from spin contamination.^[^ ^23^ ^]^ More accurate descriptions often require post‐Hartree–Fock *ab‐initio* methods,^[^ ^24^ ^]^ but their computational cost increases steeply with system size. Also, the evaluation of reaction pathways involves a series of well‐defined steps: optimizing the geometries of reactants and products, approximating the transition state (TS) geometry using various means such as the nudged elastic band method,^[^ ^25^, ^26^ ^]^ refining this TS through optimization, and performing intrinsic reaction coordinate (IRC) calculations. Even for closed‐shell reactive chemistry, quantum mechanical (QM) evaluation thus quickly becomes impractical for extensive reaction pathway explorations or high‐throughput applications.

Empirical force fields are widely used for biomolecular simulations and conformational analysis and offer a more computationally efficient alternative compared to costly QM approaches. However, their applicability to open‐shell molecules and reactive processes is limited as they lack explicit treatment of spin states.^[^ ^27^, ^28^ ^]^ A few empirical force fields, such as the ReaxFF^[^ ^29^ ^]^ and REBO^[^ ^30^ ^]^ potentials, have been created specifically for modeling reactions and can handle radical species formed during reaction. Despite their widespread use in studying dynamic evolution across diverse problems, existing parametrizations lack predictive power in mechanistic modeling of open‐shell systems.^[^ ^31^, ^32^ ^]^ Semi‐empirical methods provide a middle ground between empirical force fields and QM calculations, offering faster computations at the cost of reduced accuracy. Traditional semi‐empirical approaches like AM1^[^ ^33^, ^34^ ^]^ lack the flexibility to handle delocalized spin densities and often struggle to describe the intricate electronic effects in open‐shell species. More specialized semi‐empirical approaches have been developed for improved accuracy. Thiel's orthogonalization‐corrected methods (OMx) incorporate explicit orthogonalization corrections that improve treatment of hydrogen bonds, conformational energies, and transition states.^[^ ^34^ ^]^ These methods have demonstrated better performance for radical species, particularly for heat of formation and relative energies.^[^ ^35^ ^]^ GFN2‐xTB^[^ ^36^, ^37^ ^]^ is significantly more robust with extensive parametrization, multipole electrostatics, and dispersion corrections. However, its ability to model subtle spin‐polarization effects and diradical character is limited. Recently, AIQM1, which applies machine learning (ML) correction on top of semi‐empirical Hamiltonians based on high‐fidelity reference data, has emerged as a promising alternative.^[^ ^38^ ^]^ AIQM2 extends this framework for organic reactions, achieving accuracy beyond standard DFT for reaction energies, barriers, and TS optimization while maintaining computational cost similar to semiempirical methods.^[^ ^39^ ^]^ AIQM approaches retain the transferability and robustness of their underlying semi‐empirical foundation. However, despite being faster than QM methods, these calculations can still be computationally demanding compared to classical force fields, and their use is generally confined to initial structure generation or as prescreening tools in large‐scale workflows.

Machine learning interatomic potentials (MLIPs) have proven transformative for computational chemistry by enabling efficient, QM‐accurate approximations of PES across a wide range of problems. Models like ANI,^[^ ^40^, ^41^, ^42^ ^]^ AIMNet2,^[^ ^43^ ^]^ PaiNN,^[^ ^44^ ^]^ MACE,^[^ ^45^ ^]^ and NequIP^[^ ^46^ ^]^ have demonstrated their ability to generalize across diverse chemical spaces, supporting applications ranging from property predictions in basic organic molecules and materials to conducting molecular dynamics simulations for increasingly complex systems.^[^ ^47^, ^48^, ^49^, ^50^ ^]^ MLIPs leverage neural networks (NNs) or other ML methods to predict energies and forces directly from atomic environments, bypassing the need for explicit electronic structure calculations. However, most existing MLIPs are tailored to near‐equilibrium closed‐shell structures. Several reactive MLIPs like ANI‐1xnr^[^ ^51^ ^]^ and AIMNet2‐rxn^[^ ^52^ ^]^ have been recently developed,^[^ ^53^, ^54^, ^55^, ^56^ ^]^ but they lack the necessary descriptors for spin multiplicity or unpaired electrons for open‐shell systems. Most ML models that predict molecular properties rely only on molecular coordinates and atomic numbers as input descriptors. This hinders their generalizability to open‐shell molecules as they cannot distinguish between different spin states.

Pioneering a spin‐ and charge‐aware neural architecture, AIMNet‐NSE^[^ ^57^ ^]^ was shown to be able to predict energies for an arbitrary combination of molecular charge and spin multiplicity for neutral and charged molecules. It explicitly modelled α (spin‐up) and β (spin‐down) charges via neural spin‐charge equilibration (NSE) mechanism and enabled prediction of interpretable QM descriptors such as spin charges, Fukui indices, and vertical ionization potentials. Contrary to traditional charge equilibration approaches^[^ ^58^, ^59^, ^60^ ^]^ that apply predetermined, simple physical equations, NSE learns charge‐redistribution patterns from QM training data, thereby enabling it to capture complex, nonlinear relationships and treat spin‐polarized systems more effectively. Among other MLIPs, SpookyNet^[^ ^61^ ^]^ incorporated electronic state information by specifying the total charge and the number of unpaired electrons of a molecule. However, it does not explicitly account for atom‐resolved spin and charge densities. Alternative approaches like bpopNN^[^ ^62^ ^]^ or OrbNet^[^ ^63^ ^]^ encode information about the electronic spin‐states through electronic‐population or expectation values of different QM operators that require costly DFT or semiempirical calculations for feature extraction, making them computationally expensive. Recently, Gelžinytė et al. showed a proof‐of‐concept application of the MACE potential for predicting bond dissociation energies relevant to cytochrome P450 metabolism, with a particular emphasis on hydrogen abstraction reactions.^[^ ^64^ ^]^ The scope of this application was limited to radicals derived from homolytic cleavage of sp^3^ C─H bonds in neutral, drug‐like molecules. It did not include any charged species, nor did it account for diradicals or more complex spin topologies. Except for AIMNet‐NSE, all these MLIPs lack explicit treatment of spin, instead encoding spin information implicitly through latent features, limiting their applicability to a broader class of reactive systems and open‐shell phenomena, and leaving a critical gap in the current landscape of ML computational chemistry tools.

To address this gap, we present AIMNet2‐NSE, an MLIP explicitly designed for open‐shell systems and radical reactive chemistry. AIMNet2‐NSE is built upon the second generation of atoms‐in‐molecules neural network potential (AIMNet2),^[^ ^43^ ^]^ which currently applies to systems composed of 14 chemical elements (H, B, C, N, O, F, Si, P, S, Cl, As, Se, Br, and I) in both neutral and charged states. With an exhaustive training dataset of 20 million hybrid quantum chemical calculations, AIMNet2 combines ML‐parameterized short‐range and physics‐based long‐range terms to attain generalizability from simple organics to diverse molecules. AIMNet2 demonstrates linear scaling with system size and, with efficient GPU acceleration, can offer substantial computational advantages over *ab initio* methods as extensively demonstrated in recent benchmarks.^[^ ^43^, ^65^ ^]^ Extending on AIMNet2 and incorporating insights from AIMNet‐NSE,^[^ ^57^ ^]^ this work integrates spin‐multiplicity as an additional input for spin‐resolved learning. AIMNet2‐NSE is trained on an expanded dataset comprising the original AIMNet2 closed‐shell data, 13 million radicals, and 200K radical reaction profiles. This comprehensive training results in a model that is capable of accurate molecular property prediction across diverse charge and spin states at a fraction of the computational resources required by QM calculations. AIMNet2‐NSE represents a significant advancement in the development of transferable MLIPs by offering the computational efficiency required for high‐throughput applications while capturing the unique electronic effects of open‐shell systems and radical intermediates for reactive chemistry.

## Results and Discussion

### AIMNet2‐NSE Architecture and Training

**Figure 1:**
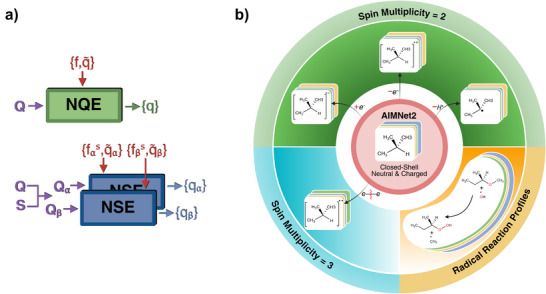
a) Architectural difference between AIMNet2 and AIMNet2‐NSE. After each message‐passing iteration, neural charge equilibration (NQE) in AIMNet2 updates atomic charges through a single channel; NSE in AIMNet2‐NSE updates atomic α and β spin‐charges through two separate channels. b) The AIMNet2‐NSE training set. It consists of the original AIMNet2 dataset spanning 14 elements, an additional 13 million open‐shell molecules in doublet and triplet spin states, and 200 000 doublet radical reaction profiles.

AIMNet2‐NSE is a message passing neural network potential that incorporates spin‐charge equilibration and explicit nonlocal Coulomb and dispersion interactions. For details of the symmetry functions, atomic environment vectors, feature vectors, and workflow, please refer to the AIMNet2 architecture.^[^ ^43^ ^]^ As shown in Figure 1a, The primary difference between AIMNet2 and AIMNet2‐NSE lies in the neural‐charge equilibration (NQE) versus NSE schemes. AIMNet2 is trained exclusively on closed‐shell neutral and charged molecules. It infers partial atomic charges from atomic feature vector representations and refines them iteratively as part of the message‐passing process. Following each charge refinement, the NQE layer is applied to ensure charge consistency across the system,

(1) $$q_{i}=\tilde{q_{i}}+\frac{f_{i}}{\sum_{j=1}^{N}f_{j}}\left(Q-\sum \tilde{q_{i}}\right)$$

where *q_i_* is the updated atomic charge for atom *i* after each message‐passing iteration, $\tilde{q_{i}}$ is the initial atomic charge, *N* is the total number of atoms in the molecule, and *Q* is the total charge of the molecule. *f_i_* serves as an atomic weight factor for redistributing atomic charges so that their total sum is equal to the specified total molecular charge. In our previous reports, we have referred to these weight factors as having a similar conceptual meaning to that of atomic Fukui functions, ∂*q_i_*/∂*Q*.^[^ ^57^ ^]^ The updated atomic charges are then integrated into the subsequent message‐passing steps, along with learned atomic features.

For AIMNet2‐NSE we adapt the full NSE methodology from Zubatyuk et al.^[^ ^57^ ^]^ In addition to atomic number, coordinates, and total charge, AIMNet2‐NSE also requires total spin multiplicity, *S*, as an input. Using *Q* and *S*, the total spin‐charges of the molecule are determined for the spin states α and β. During each message‐passing iteration, atomic spin‐charges are updated using two separate channels based on the spin state, and Equation (1) changes to,

(2) $$q_{i}^{s}=\tilde{q_{i}^{s}}+\frac{f_{i}^{s}}{\sum_{j=1}^{N}f_{j}^{s}}\left(Q^{s}-\sum \tilde{q_{i}^{s}}\right)$$

where *s*  =  α, β, and $q_{i}^{s}$ is the updated spin‐polarized atomic charges for atom *i*. In this case, $f_{i}^{s}$ resembles the spin‐polarized Fukui functions. Since the equilibrations of α and β spin‐charges are performed independently through the two channels, it can result in small spin‐polarization effects, even in closed‐shell systems. This behavior is conceptually similar to spin‐unrestricted QM calculations. We choose not to enforce zero spin‐charges for the singlet state to keep the model predictions smooth with respect to the input spin‐multiplicity. However, the overall spin conservation is preserved, as demonstrated in Figure 1a and Table S6, where the summed α and β spin‐charges exactly reproduce the specified total molecular spin charges.

Our primary strategy for training AIMNet2‐NSE was to augment the AIMNet2 dataset^[^ ^43^ ^]^ with open‐shell radical data and radical reaction profiles (Figure 1b). Doublet and triplet spin data were generated by adding or removing an electron, removing hydrogen, or unpairing electrons from all molecules in the AIMNet2 dataset that contain up to 12 atoms. ∼2M additional doublet and triplet samples were generated randomly using the same strategies for molecules containing more than 12 atoms. Unrestricted DFT single point energy calculations were performed in each case using the *ω*B97M range‐separated hybrid meta‐GGA functional^[^ ^66^ ^]^ with D3 dispersion correction^[^ ^67^ ^]^ and Becke–Johnson (BJ) damping.^[^ ^68^ ^]^ All calculations utilized the triple‐ζ valence double‐polarized basis set, def2‐TZVPP. Reference atomic charges and spin charges for training are derived from Hirshfeld population analysis^[^ ^69^ ^]^ of the unrestricted DFT electron density. Together, 13 million open‐shell molecular systems were added to the original 20 million singlet data from AIMNet2. In addition, for learning radical reaction profiles, possible doublet radicals were systematically enumerated through stepwise hydrogen abstraction from 1290 molecules with less than 50 atoms in the ConfSolv model,^[^ ^70^ ^]^ and then all possible reactions of the doublet radicals with the parent molecule were generated with the single‐ended molecular growing string method.^[^ ^71^ ^]^ Additional diversity was introduced through typical organic radicals, including methyl, ethyl, hydroxyl, and others, as radical starters, in combination with drug‐like molecules. Around 200 000 reaction profiles were selected from these reactions to add to the training dataset. See Supporting Information for detailed explanations of the methodology and level of theory.

### Reaction Energy and Barrier Height Predictions

The dataset of 5600 unique elementary radical reactions reported in Ref. [72] was used to test the new AIMNet2‐NSE interatomic potential. This dataset focuses on the main and side reactions pertinent to the polymerization of vinyl and methyl‐substituted vinyl monomers. It considers various types of species, such as initial radicals generated from RP initiators or intermediates in the autoxidation of hydrocarbons, a range of vinyl and methyl‐substituted vinyl monomers capable of RP, and 2‐Mercaptoethanol, a classical chain‐transfer agent. It also includes propagating radicals, which serve as model species for growing polymer chain‐end radicals and polymer repeating units derived from these monomers. The test set explicitly considers radical transfer to polymer repeating units as a chain‐transfer reaction and emphasizes bimolecular reactions involving radicals and closed‐shell molecules, but excludes radical‐radical recombination reactions. Additionally, selected unimolecular side reactions, particularly those influenced by molecular oxygen, are examined to account for the formation of peroxy and hydroperoxy alkyl radicals.

**Figure 2:**
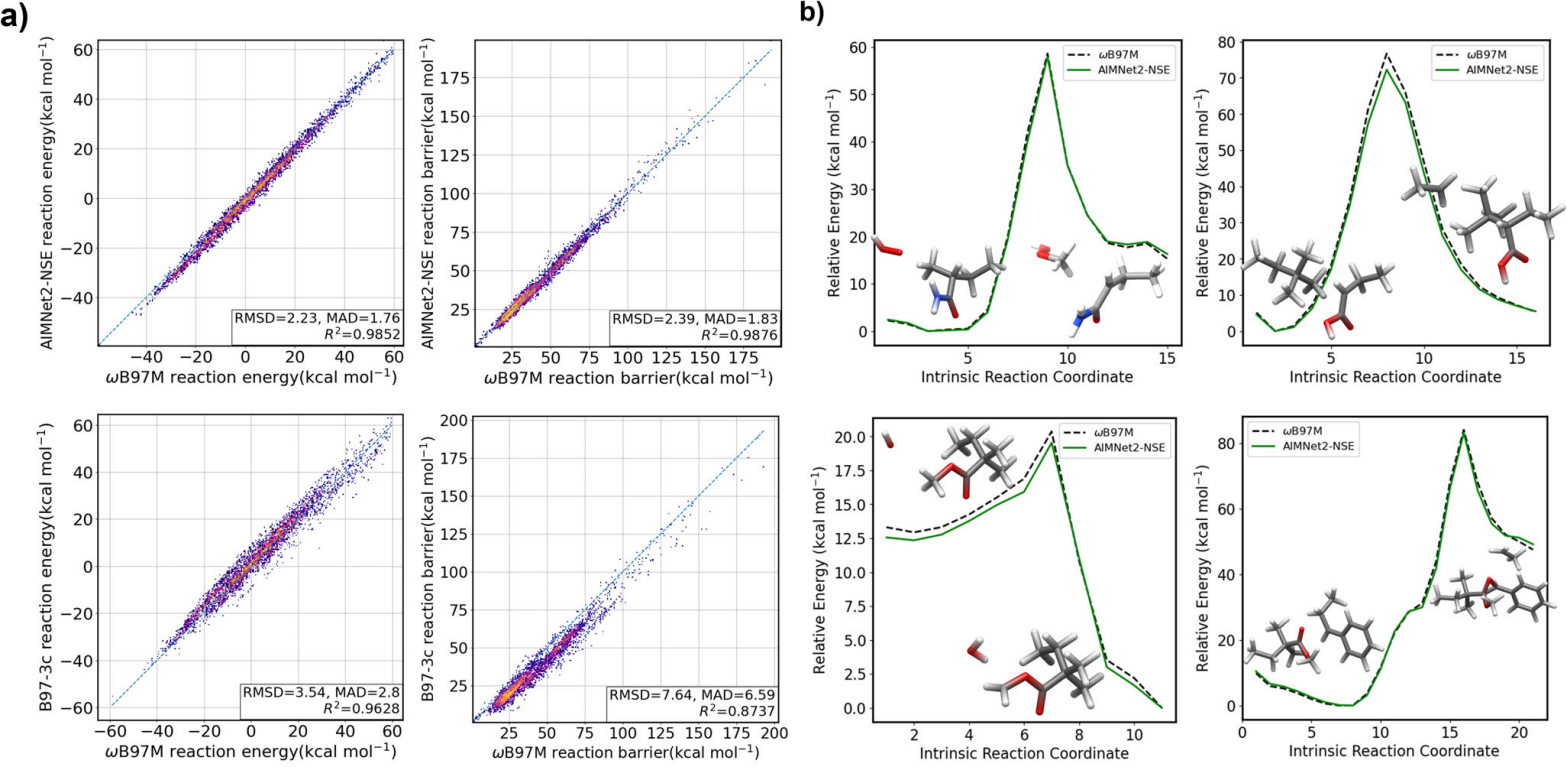
a) Comparison of AIMNet2‐NSE and B97‐3c reaction energy and reaction barrier predictions with respect to *ω*B97M‐D3(BJ)/def2‐TZVPP reference energies for all 4887 reactions from the test dataset. b) Selected reaction paths from the test dataset, computed with AIMNet2‐NSE (green) and compared with *ω*B97M‐D3(BJ)/def2‐TZVPP (dotted black) reaction profiles for radical‐initiated doublet reactions such as the formation of peroxy alkyl radicals, chain transfer reactions, and hydrogen abstraction.

AIMNet2‐NSE predicted reaction energies and reaction barriers for a subset of 4887 reactions of this test set with the same elemental coverage as AIMNet2 are compared with results obtained from composite GGA functional B97‐3c^[^ ^73^ ^]^ in Figure 2a. Reaction energies are calculated as the difference between product and reactant total energies, and reaction barriers as the difference between the TS and the reactant total energies. With a mean absolute deviation (MAD) of 1.76 kcal mol^−1^ (*R* ^2^ = 0.985), AIMNet2‐NSE reaction energy predictions are much closer to our *ω*B97M‐D3(BJ)/def2‐TZVPP reference energies compared to B97‐3c (MAD 2.80 kcal mol^−1^, *R* ^2^ = 0.963). Similarly, for reaction activation barriers, AIMNet2‐NSE outperforms B97‐3c with an MAD of 1.83 kcal mol^−1^ (*R* ^2^ = 0.988); the deviation from the reference is one‐third that of B97‐3c (MAD = 6.59 kcal mol^−1^). Hence, AIMNet2 remains a better approximation to the *ω*B97M‐D3(BJ) reference compared to B97‐3c, while also being computationally inexpensive. Given the chemical diversity and the size of the radical reaction training data set, the number of relaxed equilibrium geometries is much larger than the number of TS or off‐equilibrium structures. However, AIMNet2‐NSE maintains close accuracy for reactant, product, and TS energies for all doublet reactions in the test set. To show that the performance is reliable for the full reaction coordinate, AIMNet2‐NSE and *ω*B97M‐D3(BJ) single‐point energies are compared for four different reaction paths from the test set in Figure 2b. AIMNet2‐NSE closely approximates IRC profiles, proving its general applicability for these organic radical reactions. The overall MAD in energies across IRC profiles for all the reactions in the test set is 1.17 kcal mol^−1^.

We also verified that AIMNet2‐NSE retains the baseline accuracy of AIMNet2 for closed‐shell neutral and charged species. Figure S6 summarizes its performance on the GMTKN55^[^ ^74^ ^]^ and NCI Atlas^[^ ^75^ ^]^ benchmarks. AIMNet2‐NSE achieves comparable or slightly improved accuracy relative to AIMNet2 for basic molecular property prediction, reaction energies, and intermolecular noncovalent interactions, while showing marginally higher errors for barrier height predictions and intramolecular interactions. Overall, these results confirm that the inclusion of open‐shell data does not compromise the performance of the model for equilibrium closed‐shell systems.

### BASChem19 Benchmark

**Figure 3:**
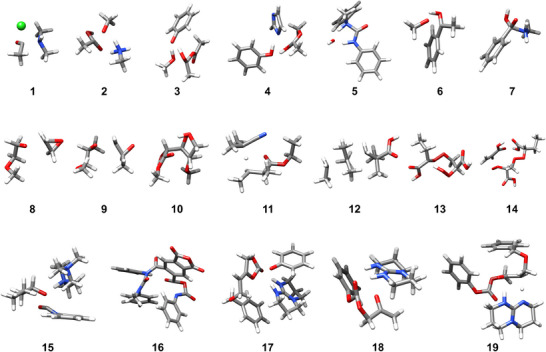
TS structures for the reactions within the BASChem19 benchmark dataset. Reactions 1–7 contain neutral polar transition states, reactions 8 and 9 are anionic, reactions 10–14 involve radical species, and reactions 15–19 are some larger complex reactions. Nitrogen: blue, oxygen: red, chlorine: green.

To assess the performance of AIMNet2‐NSE for chemical reactions found in industrial applications, we introduce a new comprehensive benchmark dataset, BASChem19, that contains the activation barriers for nineteen important radical and nonradical reactions that are often explored in polymer and chemical industry settings. An overview of the TSs included in the benchmark is shown in Figure 3. The first seven reactions consist of neutral closed‐shell, polar nucleophilic addition/substitution reactions: the nucleophilic addition of dimethylamine to acetyl chloride (1), methylamine base‐catalyzed addition of methanol to acetic acid (2), phenol‐catalyzed addition of methanol to acetic anhydride (3), phenol‐catalyzed addition of imidazole to methyl acetate (4), water shuttled addition of *N*‐methylaniline to phenyl isocyanate (5), addition of methanol to acetophenone (6), and the addition of methylamine to benzoic acid (7). The last two of these reactions are intentionally not catalyzed to also include unfavorable TS in the benchmark series, where the reaction occurs via a four‐membered ring. Reactions 8 and 9 represent the alcohol‐initiated anionic ring‐opening polymerization of ethylene oxide and propylene oxide, respectively. The next five reactions include radical species. The radical beta scission of glucose (10), 2‐cyanoprop‐2‐yl radical hydrogen abstraction from ethyl‐2‐methylbutyrate (11), addition of the terminal carbon‐centered methacrylic acid radical to tertiary carbon of 2,2‐dimethylbutane (12), intramolecular hydrogen abstraction by a peroxy radical within acrylic acid endoperoxides (13), and the terminal addition of a peroxy radical to acrylic acid (14). The last five reactions include larger, flexible species, and thus, they are representative of more complex reactions. The tertiary amine (TMEDA) catalyzed urethanization of butanol and phenyl isocyanate is given in reaction 15. Reaction 16 represents the rate‐determining step within a complex cascade of consecutive reaction steps converting pyromellitic anhydride and phenyl isocyanate to form an imide.^[^ ^76^ ^]^ In an intramolecular reaction, CO_2_ is released after the nucleophilic addition of the deprotonated amine to the anhydride unit. The last three reactions describe important steps from the organocatalytic route to endo‐vinylene carbonates from carbon dioxide‐based exo‐vinylene carbonates, including base‐catalyzed nucleophilic attack of phenol to vinylene carbonate (17), ring‐opening (18), and hydrogen transfer reactions (19). For further information, the readers are referred to Reference [77].

The BASChem19 reference structures for reactant, product, and TS were optimized with TPSS‐D3/def2‐TZVP.^[^ ^78^ ^]^ Reference activation barriers were computed with *ω*B97M‐D3(BJ)/def2‐TZVPP. The deviation in AIMNet2‐NSE activation barrier predictions, as well as in GFN2‐xTB, TPSS(D3)/def2‐TZVP, and B3LYP‐D3(BJ)/def2‐TZVP,^[^ ^79^, ^80^ ^]^ compared to *ω*B97M‐D3(BJ) reference values are shown in Table 1.

**Table 1:** Deviations in kcal mol^−1^ in activation barrier predictions with TPSS‐D3/def2‐TZVP, B3LYP‐D3(BJ)/def2‐TZVP, GFN2‐xTB, and AIMNet2‐NSE for the reactions in BASChem19 dataset. Reference energies are calculated using *ω*B97M‐D3(BJ)/def2‐TZVPP.

Reactions	TPSS‐D3/def2‐TZVP	B3LYP‐D3(BJ)/def2‐TZVP	GFN2‐xTB	AIMNet2‐NSE
1	−4.07	−1.01	−5.73	−0.97
2	−4.09	0.25	−12.63	1.83
3	−7.61	−1.19	−10.92	3.20
4	−8.88	−4.83	14.96	−1.41
5	−6.48	−2.84	5.34	3.38
6	−6.79	−0.63	−8.44	2.56
7	−6.57	−2.44	4.96	2.53
8	−9.05	−3.29	0.09	6.71
9	−5.85	−3.30	−2.61	4.07
10	−6.24	−2.76	−21.97	−0.22
11	−3.93	−1.67	−10.59	1.66
12	−13.08	−5.50	−8.97	−0.01
13	−14.41	−5.09	−45.14	−3.72
14	−10.23	−6.39	10.54	7.91
15	−5.39	−0.78	−1.32	0.37
16	−2.19	−0.89	7.81	−0.59
17	−2.05	−0.95	2.52	−0.56
18	−0.21	−1.67	8.27	0.30
19	−6.98	−3.07	−10.50	2.48
**MD**	−6.53	−2.53	−4.44	1.55
**MAD**	6.53	2.56	10.17	2.34

AIMNet2‐NSE activation barriers are found to be in better agreement with the *ω*B97M‐D3(BJ) reference (MAD 2.34 kcal mol^−1^) than the alternative DFT or semi‐empirical approaches considered. AIMNet2‐NSE significantly outperforms GFN2‐xTB (MAD 10.17 kcal mol^−1^), which is often preferred as a low‐cost screening method. As a meta‐GGA functional, TPSS consistently predicts smaller reaction barriers compared to its hybrid counterparts (MAD 6.53 kcal mol^−1^). B3LYP, a global GGA hybrid, with D3(BJ) dispersion correction, also predicts smaller reaction barriers for 18 of these reactions when compared to *ω*B97M‐D3(BJ). Since AIMNet2‐NSE is trained on *ω*B97M‐D3(BJ) energies, its prediction is closer to the reference values compared to B3LYP (MAD 2.56 kcal mol^−1^). However, prediction accuracy is the poorest for reaction 14 involving terminal addition of a peroxy radical to acrylic acid, for which both AIMNet2‐NSE and B3LYP exhibit larger deviations. This reactant has two peroxide bridges, one of which is broken in the TS. AIMNet2‐NSE underestimates the TS energy by a large margin. Both reactions 13 and 14 suggest that we may require additional sampling and training for peroxide compounds. AIMNet2‐NSE prediction errors are also higher for the anionic ring‐opening polymerizations of ethylene oxide (reaction 8) and propylene oxide (reaction 9), each containing a three‐membered ring either in the reactant or in the TS. However, it reproduces reference barrier energies for the neutral five‐membered ring‐opening transformation of exo‐vinylene carbonates to endo‐vinylene carbonates, within 0.30 kcal mol^−1^ (reaction 18). Overall, AIMNet2‐NSE performs well for both smaller neutral reaction intermediates and larger, more complex reactions.

### RP Reaction Case Studies

Here, we consider RP initiation or termination reactions from Reference 7 to show proof‐of‐concept applications of AIMNet2‐NSE for industrial purposes. Both geometries and relative energies along each reaction coordinate are independently calculated with *ω*B97M‐D3(BJ)/def2‐TZVPP and AIMNet2‐NSE. Energy‐only comparisons on DFT geometries are included in the Supporting Information.

**Figure 4:**
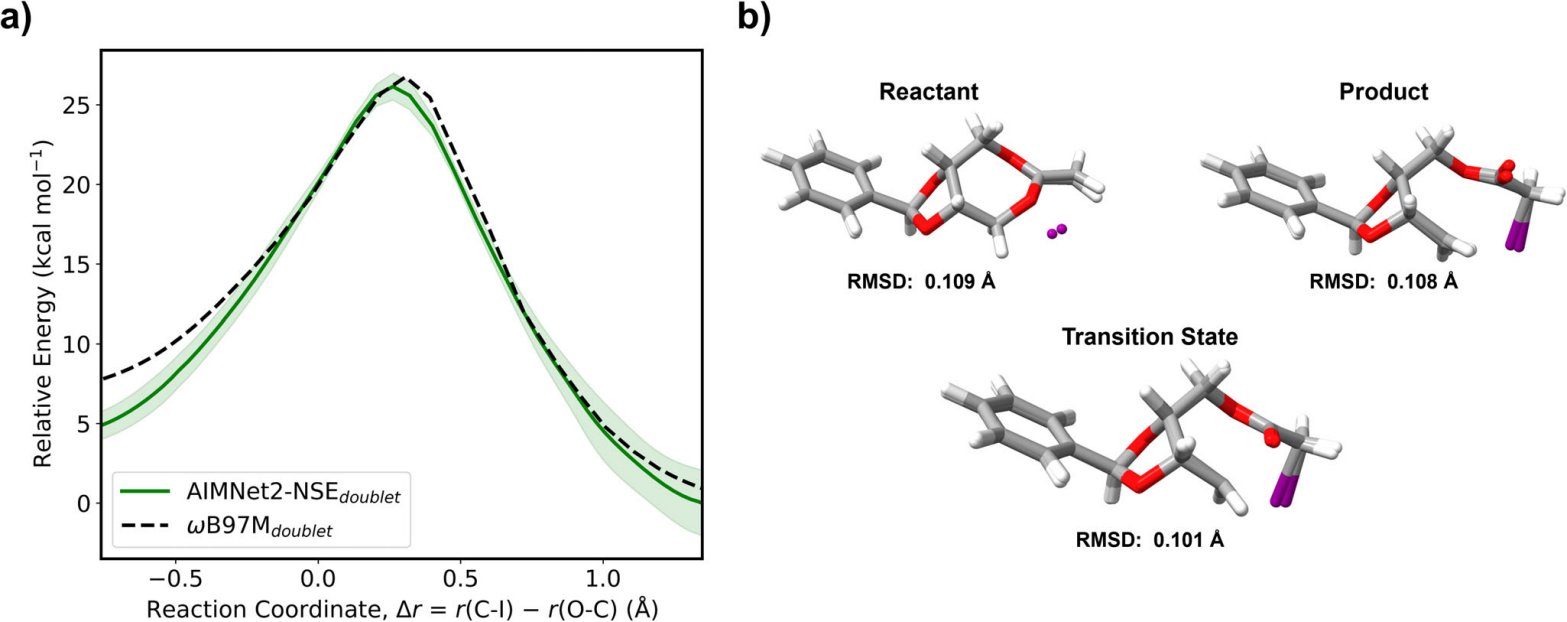
a) A comparison of the IRCs obtained from AIMNet2‐NSE (green) and *ω*B97M‐D3(BJ) (dotted black) for the iodine‐mediated ring opening polymerization of 2‐methylene‐1,3‐dioxepane. Trajectories were calculated with an ensemble of four AIMNet2‐NSE models; mean and standard deviations are shown here. b) Structural overlaps between *ω*B97M‐D3(BJ) and AIMNet2‐NSE geometries and the corresponding RMSDs for reactant, product, and TS for the same reaction. Oxygen: red, iodine: purple.

A well‐known RP reaction is the ring‐opening polymerization of cyclic monomers, similar to reactions 8, 9, and 18 in the BASChem19 database. The ring‐opening polymerization of 2‐methylene‐1,3‐dioxepane occurs in the presence of iodine, and both the reactant and product contain a single unpaired electron. An independent TS search is performed using the AIMNet2‐NSE plugin with Pysisyphus.^[^ ^81^ ^]^ The IRCs from benchmark *ω*B97M‐D3(BJ) and AIMNet2‐NSE for this ring‐opening polymerization reaction are shown in Figure 4a. The activation barrier obtained from AIMNet2‐NSE ensemble models (27.67 kcal mol^−1^) is a slight underestimate compared to the DFT benchmark (27.84 kcal mol^−1^). The normalized IRC remains reasonably accurate throughout the rest of the reaction coordinates. The predicted reaction energy is just 1.65 kcal mol^−1^, somewhat comparable to *ω*B97M‐D3(BJ) (3.44 kcal mol^−1^). The RMSD for the AIMNet2‐NSE reactant, product, and TS structures obtained from Pysisyphus IRC are shown in Figure 4b. AIMNet2‐NSE structures closely approximate those obtained from DFT. Beyond its predictive accuracy, AIMNet2‐NSE offers substantial computational efficiency. For this simple example, locating and optimizing the TS and computing the IRC with *ω*B97M‐D3(BJ) requires approximately 2944 CPU core hours, whereas the equivalent workflow carried out entirely with AIMNet2‐NSE using gradients obtained via automatic differentiation takes only a few seconds, resulting in a 10^5^ times computational speed‐up.

To evaluate whether these capabilities extend to larger polymeric systems, we performed a size‐consistency analysis for the radical propagation of methyl acrylate across chain lengths *n* = 2–5 (Figure S5 and Table S1). AIMNet2‐NSE models were able to predict stable activation barriers and reaction energies across different chain lengths, indicating that the NSE remains robust beyond the scale of the training set.

Common RP reactions are often accompanied by a change in spin state, like simple bond‐dissociation of a neutral molecule resulting in two radicals. While many MLIPs focus on smooth interpolation between equilibrium singlet and dissociated biradical triplet state,^[^ ^64^ ^]^ spin‐resolved PES are hardly explored. Our training data did not include singlet‐to‐triplet bond‐dissociation, but the inherent design of the NSE block gives us the flexibility to evaluate both singlet and triplet PES across the bond‐breaking coordinates. While standard DFT can compute singlet and triplet surfaces, accurately capturing the physics of spin‐crossing events would require more sophisticated approaches. Here, we evaluate the performance of AIMNet2‐NSE within these known DFT constraints. The first example we consider is the dissociation of silylated benzopinacol, a robust RP initiator that benefits from steric protection, stabilizing the resulting radicals and offering superior handling and storage stability.^[^ ^7^ ^]^

**Figure 5:**
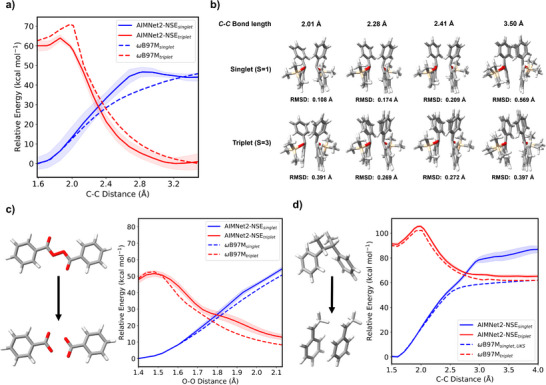
a) AIMNet2‐NSE optimized energies and comparison with *ω*B97M‐D3(BJ) for singlet and triplet PES scan along the bond‐coordinates for homolytic bond‐dissociation of silylated benzopinacol. b) Overlaps between *ω*B97M‐D3(BJ) and AIMNet2‐NSE predicted structures at a few selected coordinates along the PES and their RMSD for both spin states. c) Singlet and triplet PES for both *ω*B97M‐D3(BJ) and AIMNet2‐NSE geometries along the bond coordinates for the dissociation of benzoyl peroxide. The lowest energy from each method is shifted to 0 kcal mol^−1^ for both spin states. d) Unrestricted DFT singlet and triplet PES comparison to AIMNet2‐NSE for the formation of the styrene radical. *ω*B97M‐D3(BJ): dotted lines, AIMNet2‐NSE: solid lines, oxygen: red, silicon: beige. Trajectories were calculated with an ensemble of four AIMNet2‐NSE models; mean and standard deviations are depicted here.

Figure 5a presents the singlet and triplet PESs computed by AIMNet2‐NSE alongside *ω*B97M‐D3(BJ) trajectories, and Figure 5b depicts the RMSD for four off‐equilibrium geometries for the radical decomposition of silylated benzopinacol. AIMNet2‐NSE, despite not being trained to predict bond‐dissociation trajectories, approximates the PESs with reasonable accuracy. The predicted bond‐dissociation energy for independent optimization is 37.84 kcal mol^−1^ (AIMNet2‐NSE) versus 41.38 kcal mol^−1^ (DFT). Structural comparisons reveal that AIMNet2‐NSE maintains a low RMSD of 0.07 Å from the DFT‐optimized undissociated structure. As dissociation progresses, RMSD increases to 0.40 Å (when the separation between the two radicals is around 3.5 Å), indicating some deviation in complex conformational spaces that may result from algorithmic divergence associated with geometry optimization as elaborated in Ref. 64. A robust conformer search should be conducted to obtain a closer approximation to DFT. Both energy and structural mismatches are larger for the undissociated structure in the triplet state and the dissociated radicals in the singlet state, which suggests that AIMNet2‐NSE training data could benefit from further sampling of these high‐energy, unphysical regions of the PES. Nevertheless, AIMNet2‐NSE provides sufficiently accurate geometries to serve as initial guesses for more expensive refinement with high‐level QM calculations.

Another example is dibenzoyl peroxide, or benzoyl peroxide, which is a widely utilized radical initiator in industrial applications. It undergoes homolysis of its weak O─O bond to generate benzoyloxy radicals. With restricted, closed‐shell, singlet DFT, a bond scan reveals a rearrangement around 2.1 Å, forming two distinct closed‐shell products (Figure S12). In contrast, AIMNet2‐NSE singlet calculation predicts a qualitatively different PES; as we stretch the O─O bond, one of the dissociating oxygen atoms recombines with the second oxygen atom on the other fragment, effectively returning to the original undissociated molecule. This reflects the learned pattern of AIMNet2‐NSE from the training data distribution rather than an intrinsic capability of the model architecture. Figure 5c shows good agreement between the *ω*B97M‐D3(BJ) and AIMNet2‐NSE PESs along the O─O bond dissociation coordinates up to 2.1 Å. The approximate bond dissociation energy computed with *ω*B97M‐D3(BJ) is 36.53 kcal mol^−1^, whereas AIMNet2‐NSE yields a value of 37.13 kcal mol^−1^.

One approach to prevent unphysical radical recombination during singlet state bond dissociation is to use unrestricted DFT calculations, which can yield more reasonable geometries but at the cost of potential spin contamination. For comparison, we perform unrestricted Kohn‐Sham DFT (UKS) calculations for the decoupling of styrene dimer in both singlet and triplet spin states. Figure 5d shows the PESs obtained from UKS *ω*B97M‐D3(BJ) calculation and AIMNet2‐NSE optimization. Calculated reaction energies for the completely dissociated species are 67.61 kcal mol^−1^, from DFT and 67.47 kcal mol^−1^, from AIMNet2‐NSE, indicating close approximation. While AIMNet2‐NSE singlet PES remains stable throughout the bond‐dissociation, restricted DFT calculations will undergo radical rearrangement and recombination in less than 2 Å. Singlet AIMNet2‐NSE exhibits the typical overestimation of bond‐dissociation energy observed with restricted closed‐shell DFT compared to unrestricted solutions.^[^ ^82^ ^]^ Thus, independent equilibration of α and β spin‐channels in AIMNet2‐NSE enables robust treatment of spin‐polarized systems. However, the model's predictive accuracy is ultimately constrained by the limited representation of unphysical, high‐energy regions of the PES in the training data.

For dissociation of benzoyl peroxide, starting from the same reactant geometry, the RMSD between AIMNet2‐NSE and *ω*B97M‐D3(BJ) structures is 0.05 Å. Reoptimizing the *ω*B97M‐D3(BJ) structure with AIMNet2‐NSE reduces the RMSD to as low as 0.01 Å. This value increases throughout the PES, reaching 0.15 Å for the dissociated products. The RMSD for styrene dimer optimized from *ω*B97M‐D3(BJ) geometry using AIMNet2‐NSE is 0.02 Å, and it increases to 0.11 Å for the dissociated styrene radical pair. Coming back to computational efficiency, a simple optimization task starting from the same geometry results in a 10^5^x speedup with AIMNet2‐NSE. For silylated benzopinacol (74 atoms), a 14‐point coordinate scan using restricted DFT for the singlet state requires approximately 24 h on 128 CPU cores, whereas the triplet state demands around 41 h. In contrast, AIMNet2‐NSE completes the same task within a few seconds of CPU time, again offering an approximate 5 or 6 orders of magnitude computational speedup. PES scans of benzoyl peroxide and styrene dimer using AIMNet2‐NSE show similar computational speed‐ups.

For our final example, we consider AIBN, azobisisobutyronitrile, a very important azoinitiator, frequently used in several RP initiation reactions in the polymer industry, such as reaction 11 from the BASChem19 dataset. When heated, AIBN undergoes homolytic cleavage at the weak C─N bonds, generating nitrogen gas and two carbon‐centered 2‐cyanoprop‐2‐yl radicals in a concerted reaction. Modeling AIBN's decomposition poses challenges for QM methods, as the reaction involves concerted bond‐breaking and transitions between electronic states. These nonadiabatic transitions necessitate modeling across multiple PES with the reaction barrier approximated by the minimum energy crossing point (MECP) between two spin states.^[^ ^83^, ^84^, ^85^ ^]^ While multi‐reference methods are ideal for accurately capturing such phenomena, they are computationally intensive.^[^ ^86^ ^]^ AIBN decomposition is an excellent test case for methods like AIMNet2‐NSE, which can handle these calculations without SCF convergence issues that can plague traditional DFT calculations.

**Figure 6:**
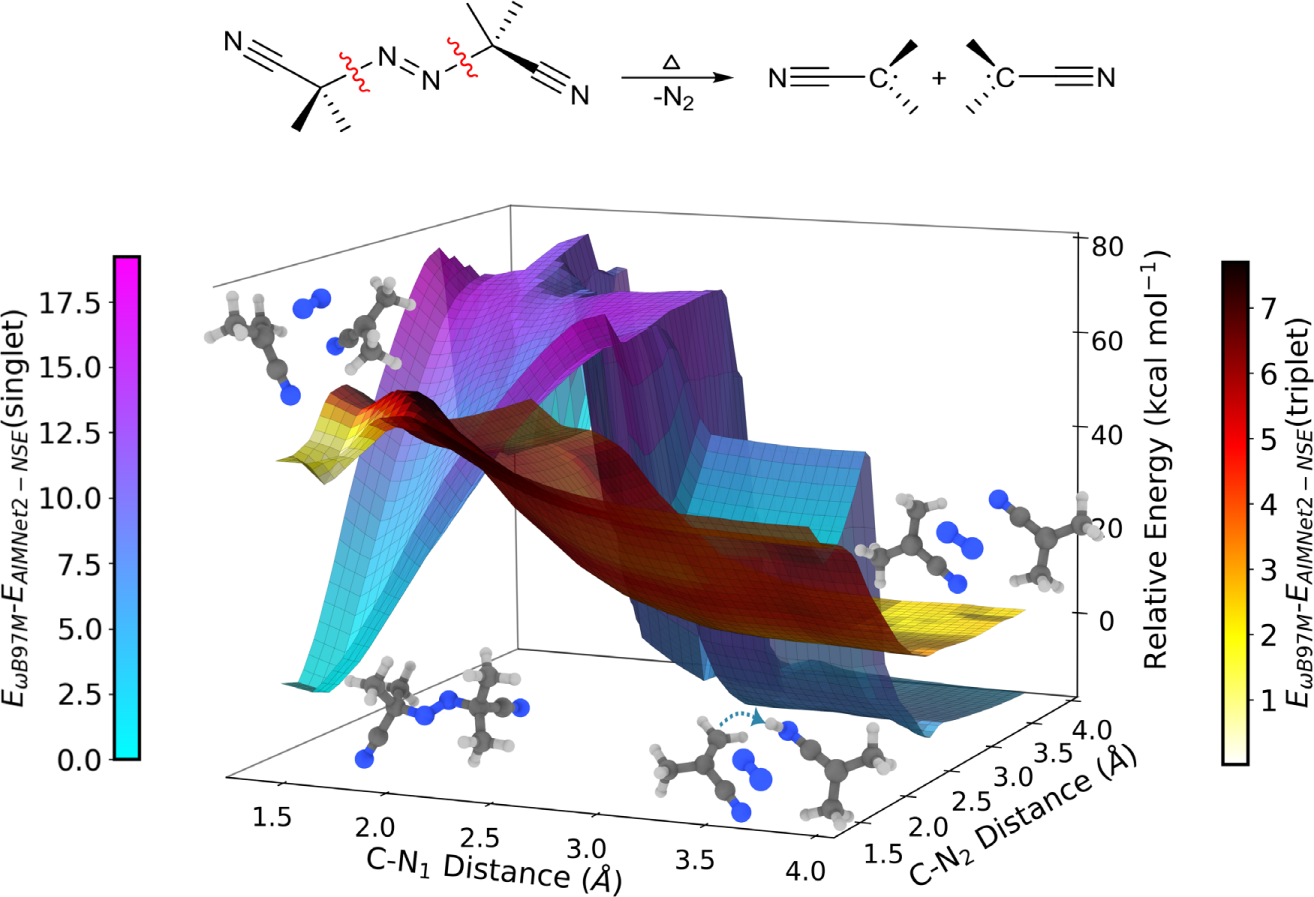
Two‐dimensional (2D) PES scan along each C‐N coordinate for the thermal decomposition of AIBN with the dissociation of N_2_. Singlet and triplet energies along the PES are calculated with AIMNet2‐NSE for B3LYP geometries, and errors are evaluated based on *ω*B97M‐D3(BJ) single‐point energies on the same geometries. This color gradient represents the MAD in energy between *ω*B97M‐D3(BJ) and AIMNet2‐NSE. The cyan–magenta color map represents the singlet PES, and the red–yellow color map represents the triplet PES.

To assess AIMNet2‐NSE's performance, we conducted PES scans along the two C─N bond coordinates of AIBN for both singlet and triplet spin states. Geometries were optimized using B3LYP‐D3(BJ)/def2‐TZVP, and single‐point energies were calculated for them with both *ω*B97M‐D3(BJ)/def2‐TZVPP and AIMNet2‐NSE. The PES obtained from the two‐dimensional (2D) scan and the corresponding deviations in energy predictions from AIMNet2‐NSE compared to the reference are shown in Figure 6. As both C─N bonds elongate, the triplet PES approaches a TS leading to the formation of nitrogen gas and two radicals. This transformation proceeds symmetrically along both C─N bonds. The singlet PES initially stays stable as the bonds elongate and intersect the triplet surface near the triplet transition region. Beyond this point, however, the singlet surface leads to a rearranged product via hydrogen migration, resulting in two dissociated closed‐shell species more stable compared to AIBN or the diradical products.

AIMNet2‐NSE accurately captures the triplet PES, with MAD in energy of approximately 1–2 kcal mol^−1^ for both reactant and products. In regions near the singlet‐triplet crossing, the error increases to 6–7 kcal mol^−1^. The difference becomes more pronounced, 15–17 kcal mol^−1^, near the peak of the singlet PES. These deviations are expected, as AIMNet2‐NSE is not trained to predict such high‐energy unphysical parts of the PES. Without entropic and thermal consideration, the reaction energy is predicted to be a mere −0.75 kcal mol^−1^ with *ω*B97M‐D3(BJ), −13.34 kcal mol^−1^ with B3LYP, and 1.68 kcal mol^−1^ with AIMNet2‐NSE. Determining the approximate crossing energy is again questionable, as the singlet surface leads to different products. Concentrating on the region between 1.5 and 2.5 Å, the closest symmetric contact between the two surfaces happens around 2.05 Å (Figure S15 and Table S9). With AIMNet2‐NSE, the symmetric point of contact occurs at a slightly elongated distance of 2.21 Å (Figure S16 and Table S10). The approximate crossing energy between the singlet and triplet surfaces at this location is 24.47 kcal mol^−1^ with B3LYP‐D3(BJ)/def2‐TZVPP and 38.31 kcal mol^−1^ with *ω*B97M‐D3(BJ)/def2‐TZVPP. With AIMNet2‐NSE, the predicted crossing energy is 33.31 kcal mol^−1^. Experimental activation energy values are close to 30.8 kcal mol^−1^.^[^ ^87^, ^88^ ^]^ Although this crossing energy should not be compared to the true activation barrier, and more accurate MECP calculations should be performed, this simple illustration proves that AIMNet2‐NSE can yield reasonable approximations for practical applications. The geometry around the crossing region can also be used as a starting guess for more accurate MECP evaluation. It's important to note that AIMNet2‐NSE, trained primarily on equilibrium structures, may not fully capture the complexities of off‐equilibrium or rearranged states, such as those involving hydrogen migration observed in the singlet PES of AIBN. These findings highlight both the practical utility of AIMNet2‐NSE and opportunities for further refinement of the AIMNet2 architecture and related MLIP frameworks.

## Conclusion

In this work, we introduced AIMNet2‐NSE, a neural network potential tailored specifically for reactive open‐shell systems with an arbitrary combination of charge and spin multiplicity. AIMNet2‐NSE represents a significant advancement in the computational modeling of open‐shell systems and radical reactive chemistry with MLIPs. By integrating NSE into a transferable machine‐learned potential, we have created a model that maintains near‐QM accuracy while dramatically reducing computational costs compared to traditional QM methods. By distinguishing between different spin states, AIMNet2‐NSE enables accurate modeling of the complex electronic environments essential for predicting radical reaction mechanisms and spin‐crossing events.

We also introduced BASChem19 benchmark—a dataset for evaluating computational chemistry methods on industrially significant reactions. What makes these reactions particularly valuable to the chemical industry is their direct application in producing commercially important materials and intermediates. For instance, the RP reactions featured in the dataset emphasize that nearly half of all industrially manufactured polymers are obtained through these processes. Moreover, nucleophilic additions are fundamental to pharmaceutical synthesis and specialty chemical production. Rather than focusing on simplified model reactions, BASChem19 captures the complexity of real industrial processes, including challenging transition states and multiple reaction pathways, providing a realistic test of how novel computational methods might accelerate innovation.

Our extensive evaluations across diverse radical reactions, including industrial polymerization processes and the BASChem19 benchmark, demonstrate the model's robust transferability and remarkable prediction capabilities. AIMNet2‐NSE consistently outperforms established computational methods, including B97‐3c and GFN2‐xTB. Despite these achievements, several challenges remain in the field of MLIPs for open‐shell systems. In its current form, AIMNet2‐NSE is not directly applicable to open‐shell singlets as well as bulk or condensedphase systems and would require additional considerations for periodic effects.^[^ ^89^ ^]^ The accurate representation of TS, particularly for reactions far from equilibrium configurations, still requires careful data generation and targeted training protocols. As we continue to refine AIMNet2‐NSE, future work will focus on expanding the training dataset to encompass more diverse reaction environments, broader elemental and spin state coverage, and multi‐reference electronic structure calculations.

One critical direction would be expanding the AIMNet2‐NSE elemental coverage beyond organic elements to include transition metals, which play essential roles in catalysis and metalorganic radical chemistry.^[^ ^90^ ^]^ This expansion would enable modeling of complex systems like metalloenzymes, organometallic catalysts, and metal‐organic frameworks that frequently involve radical intermediates and multiple spin states. Another significant direction involves improving the accuracy of TS predictions through targeted data generation strategies. While AIMNet2‐NSE performs well on reaction energetics, developing specialized approaches for sampling TS regions and rare reactive events would enhance its ability to model reaction kinetics accurately. Integration with enhanced sampling techniques such as metadynamics or replica exchange methods could address challenges in barrier height predictions, particularly for complex reactions.

Perhaps the most exciting prospect lies in applying AIMNet2‐NSE to high‐throughput virtual screening of radical reactions for industrial applications. The model's computational efficiency makes it well‐suited for exploring vast chemical spaces to identify promising candidates for polymerization catalysts, radical initiators, or novel materials with tailored properties. Coupling AIMNet2‐NSE with active learning and automated reaction pathway discovery algorithms could revolutionize areas of polymer design, where understanding RP mechanisms remains challenging.

## Supporting Information

The authors have cited additional references within the Supporting Information.^[^ ^91^, ^92^, ^93^, ^94^, ^95^, ^96^, ^97^, ^98^, ^99^, ^100^, ^101^, ^102^, ^103^, ^104^, ^105^, ^106^, ^107^ ^]^

## Conflict of Interests

The authors declare no conflict of interest.

## Supporting information



Supporting Information

Supporting Information

## Data Availability

The BASChem19 benchmark dataset is available at https://figshare.com/s/988db758cceb66f220c1. The pre‐trained AIMNet2‐NSE models and the code to reproduce this study are available in GitHub at https://github.com/isayevlab/aimnetcentral/.
