# Supplementary material for: AIMNet2‐NSE: A Transferable Reactive Neural Network Potential for Open‐Shell Chemistry
Source: Angew Chem Int Ed Engl. 2025 Dec 16;65(5):e16763. doi: 10.1002/anie.202516763 (PMC12851018; doi:10.1002/anie.202516763)
Supplement: Supplementary file 1 — Supporting Information [file ANIE-65-e16763-s002.pdf]

Supplementary Materials for

AIMNet2-NSE: A Transferable Reactive Neural Network Potential for Open-Shell Chemistry

Bhupalee Kalita+,[a], Roman Zubatyuk+,[a], Dylan M. Anstine[b], Maike Bergeler[c], Volker Settels[c], Conrad Stork[c], Sebastian Spicher*,[c], Olexandr Isayev*,[a,d]

+ *These authors contributed equally*

* *Corresponding authors*

[a] Dr. B. Kalita, Dr. R. Zubatyuk, Dr. O. Isayev
Department of Chemistry
Carnegie Mellon University
Pittsburgh, PA, 15213, United States
E-mail: olexandr@olexandrisayev.com

[b] Dr. D. M. Anstine
Department of Chemical Engineering and Materials Science
Michigan State University
East Lansing, MI 48824, United States

[c] Dr. M. Bergeler, Dr. V. Settels, Dr. C. Stork, Dr. S. Spicher
BASF SE
Carl-Bosch Straße 38, 67056 Ludwigshafen am Rhein, Germany
E-mail: sebastian.spicher@basf.com

[d] Dr. O. Isayev
Department of Materials Science and Engineering
Carnegie Mellon University
Pittsburgh, PA, 15213, United States

Table of Contents

1. Computational Methods 5
   a) Training and Test Data Generation 5
   b) BASChem19 7
   c) Radical Polymerization Reaction Case Studies 7
2. Training Protocol 8
3. AIMNet2-NSE Geometry Optimization 9
4. Geometry-Based Similarity and Scaffold Analysis 9
5. Size Consistency Analysis for Radical Polymerization Reactions 10
6. Additional Benchmarks and Results 12
   a) GMTKN55 and NCI Atlas 12
   b) Ionization potentials and electron affinity benchmarks 14
   c) Evaluation of atomic and spin charges: 16
   d) Treatment of dications and quintets 19
   e) Miscellaneous 20

Table of Figures

Figure S1. Scheme to generate organic doublet radicals via homolytic dissociation of all covalent bonds between hetero atoms and hydrogen. The resulting radicals undergo a reaction with their closed-shell parent molecule the radical was derived from. 5
Figure S2. A set of radicals used in training data generation that react with the list of drug-like molecules to generate further radical species. 5
Figure S3. Top: An example of how propagating radicals are formed from a vinyl monomer. Bottom: Example to demonstrate the polymer repeating unit model generation from the corresponding (vinyl) monomer. 6
Figure S4. The grwoing-chain plymerization of methyl acrylate 10
Figure S5. Size consistency analysis for methyl acrylate radical polymerization. Activation energies ($E_{Activation}$, upper cluster) and reaction energies ($E_{Reaction}$, lower cluster) are shown as a function of polymer chain length (n = 2-5 monomer units). All four AIMNet2-NSE models (Model 1-4) are compared with TPSS/def2-TZVP reference calculations. Size consistency would be indicated by horizontal lines (constant energy regardless of chain length). 10
Figure S6. Performance of several DFT hybrid and GGA functionals, GFN2-xTB, AIMNet2, and AIMNet2-NSE on the GMNTK55 benchmark (closed-shell, top) and Non-Covalent Interaction (NCI) Atlas benchmark (closed-shell, bottom). HB300SPX×10 - Hydrogen bonding extended to S, P, and halogens; HB375×10 - Hydrogen bonding in organic molecules; IHB100×10 - Ionic hydrogen bonds in organic molecules; R739×5 - Repulsive contacts in an extended chemical space; SH250×10 - Sigma-hole interactions; D442×10 - London dispersion in an extended chemical space 12
Figure S7. (a) Benzene: Total atomic charges obtained from Hirshfeld population analysis of *ω*B97M-D3(BJ) electron density compared with AIMNet2-NSE predictions. (b) Dibenzo[a,f]pentalene; a ground-state open-shell singlet molecule 16
Figure S8. Paraquat or *N*,*N*′-dimethyl-4,4′-bipyridinium dication ($[(C_6H_7N)_2]^{2+}$), also known as methyl viologen. Paraquat is a highly toxic organic compound and is widely used as herbicide. 19
Figure S9. Comparison of AIMNet2-NSE and *ω*B97M-D3(BJ)/def2-TZVPP energies for the homolytic bond-dissociation of molecular oxygen in both triplet and quintet states 19
Figure S10. Iodine-mediated ring opening polymerization of 2-methylene-l,3-dioxepane. A comparison of doublet IRCs obtained from AIMNet2-NSE on DFT geometries, and *ω*B97M-D3(BJ)/def2-TZVPP energies. Trajectories were calculated with an ensemble of four AIMNet2-NSE models, and mean, and standard deviation are depicted here 20
Figure S11. AIMNet2-NSE energies evaluated on the DFT geometries and comparison with corresponding DFT values (*ω*B97M-D3(BJ)/def2-TZVPP) for the homolytic bond-dissociation of silylated benzopinacol, in both singlet and triplet spin states. Trajectories were calculated with an ensemble of four AIMNet2-NSE models and mean, and standard deviations are depicted here. 20
Figure S12. AIMNet2-NSE energies evaluated on the DFT geometries and comparison with corresponding DFT values (*ω*B97M-D3(BJ)/def2-TZVPP) for dissociation of dibenzoyl peroxide, for both spin states. A potential surface energy scan done for the singlet state undergoes rearrangement around 2.1 Å to form two different closed-shell products. Trajectories were calculated with an ensemble of four AIMNet2-NSE models. 21
Figure S13. AIMNet2-NSE energies evaluated on the DFT geometries and comparison with corresponding unrestricted DFT values (*ω*B97M-D3(BJ)/def2-TZVPP) for the homolytic bond-dissociation of styrene, for both S=1 and S=3 spin states. Trajectories were calculated with an ensemble of four AIMNet2-NSE models. 21
Figure S14. A 2D scan of the Thermal decomposition of AIBN with the dissociation of N_2 as calculated by DFT. Single point energies are calculated with *ω*B97M-D3(BJ)/def2-TZVPP on B3LYP geometries. The AIMNet2-NSE predictions across the two surfaces are shown in Figure 6. 22
Figure S15. The energy difference between the singlet and triplet surfaces for thermal decomposition of AIBN with the dissociation of N_2 as calculated by DFT. Single point energies are calculated with *ω*B97M-D3(BJ)/def2-TZVPP on B3LYP geometries. 23
Figure S16. The energy difference between the singlet and triplet surfaces for thermal decomposition of AIBN with the dissociation of N_2 as calculated with AIMNet2-NSE on B3LYP geometries 23
Figure S17. Representative Pysisyphus YAML configuration file for geometry optimization using AIMNet2-NSE analytical Hessians (requires aimnet2pysis plugin: https://github.com/isayevlab/aimnetcentral) 28
Figure S18. Complete reaction pathway characterization workflow using AIMNet2-NSE: Geometry optimization → Growing String → TS optimization → IRC → endpoint optimization (requires aimnet2pysis plugin: https://github.com/isayevlab/aimnetcentral) 29

Table of Tables

Table S1.(a) Activation energies (kcal/mol) for methyl acrylate radical polymerization as a function of chain length, **(b)** Reaction energies (kcal/mol) for methyl acrylate radical polymerization as a function of chain length. 11
Table S2. Mean absolute errors (MAE) in energy in kcal/mol for GMTKN55 subsets (closed-shell, charged)...... 13
Table S3. Deviations in kcal/mol in ionization potential predictions calculated with *ω*B97X-D3 and AIMNet2-NSE for molecules in the G21IP dataset 14
Table S4. Deviations in kcal/mol in electron affinity predictions calculated with *ω*B97X-D3 and AIMNet2-NSE for molecules in the G21EA dataset 15
Table S5. Total atomic charges and spin charges obtained from Hirshfeld population analysis of *ω*B97M-D3(BJ) electron density for dibenzo[a,f]pentalene in singlet, triplet and open-shell singlet spin states............................. 17
Table S6. Total atomic charges and spin charges predicted with AIMNet2-NSE for dibenzo[a,f]pentalene in singlet and triplet spin states. 17
Table S7. Comparison between *ω*B97M-D3(BJ)/def2-TZVPP and AIMNet2-NSE activation barriers and reaction energies obtained from thermochemical analysis for the ring-opening polymerization of 2-methylene-1,3-dioxepane. AIMNet2-NSE predictions are made for with a single model instead of an ensemble of four models. All values are in kcal/mol. 22
Table S8. The final dissociated radical geometries obtained from *ω*B97M-D3(BJ)/def2-TZVPP and each of the four AIMNet2-NSE models for the dissociation of dibenzoyl peroxide and styrene dimer 24
Table S9. Five closest points of contact between the singlet and triplet PES calculated with *ω*B97M-D3(BJ)/def2-TZVPP for thermal decomposition of AIBN 24
Table S10. Five closest points of contact between the singlet and triplet surfaces calculated with AIMNet2-NSE for thermal decomposition of AIBN 25
Table S11. Comparison of vibrational frequencies calculated with *ω*B97M-D3(BJ)/def2-TZVPP and AIMNet2-NSE for 2-methylene-1,3-dioxepane. Frequency analysis was performed on the same optimized geometry. AIMNet2-NSE predictions were obtained using a single model rather than an ensemble of four models. All frequencies are in cm^{-1}..25
Table S12. Energies for the singlet and triplet 2D scan of AIBN decomposition with the dissociation of N_2 as calculated by *ω*B97M-D3(BJ)/def2-TZVPP and AIMNet2-NSE on B3LYP-D3(BJ)/def2-TZVP geometries. 2D scan was performed in four equal parts and each part considered 81 geometries in both spin states. The AIMNet2-NSE predictions across the two surfaces are shown in Figure 6……………………………………………………29

1. Computational Methods

a) Training and Test Data Generation

For learning radical reaction profiles, we selected 1,290 molecules with less than 50 atoms from the recently developed ConfSolv model[1]. We systematically enumerated possible doublet radicals through stepwise hydrogen abstraction from a molecule (Figure S1). All possible reactions of the generated radical with its closed-shell unsubtracted parent molecule were initiated with a workflow based on the single-ended molecular growing string method (mGSM)[2] as described in Ref.[3]. With a slight deviation from the procedure described in Ref.[3], reaction paths were calculated with GFN2-xTB[4,5]. The total number of bonds that were allowed to be either formed or broken was set to two. The reaction path was discarded, and only the optimized endpoints, reactants, and products, were chosen as reference structures. This process generated over 1.6 million organic radical reactions for the training data.

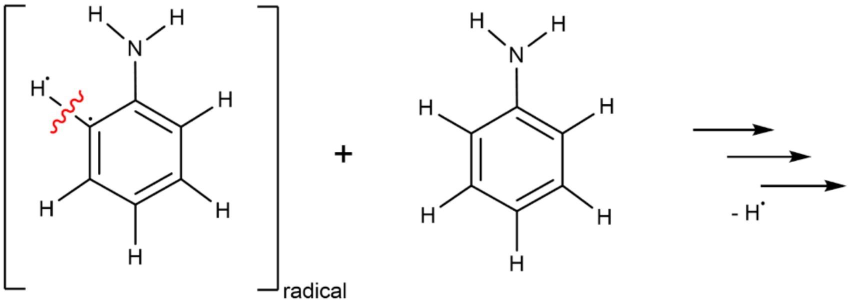

Figure S1. Scheme to generate organic doublet radicals via homolytic dissociation of all covalent bonds between hetero atoms and hydrogen. The resulting radicals undergo a reaction with their closed-shell parent molecule the radical was derived from.

To increase the diversity of the training data further, typical organic radicals, including methyl, ethyl, hydroxyl, hydroperoxyl, alkoxy, methyl peroxy, cyanoprop-2-yl, ester alkoxy, ester peroxy, benzoyl, aminyl, and phenoxy radicals, were also used as input for mGSM (Figure S2). The modified procedure from Ref.**[3]** was also applied here, and all possible reactions of all radical-monomer combinations, in which a maximum of two bonds are formed or broken, were calculated using the single-ended mGSM method at the GFN2-xTB**[4,5]** level of theory. Endpoints were optimized at the same level of theory and added to the training data. Thereby, the training data set was expanded by a further 110,000 structures.

A subset of around 200,000 reactant-product pairs was taken from the above-generated double-reaction dataset and added to the training set. Reaction profiles were sampled with climbing image nudged elastic band method[6] and the initial seed AIMNet2-NSE model, and samples were selected based on QBC uncertainty. Single-point energies were recalculated for reactants, products, and corresponding reaction profiles with ωB97M-D3(BJ)/def2-TZVPP.

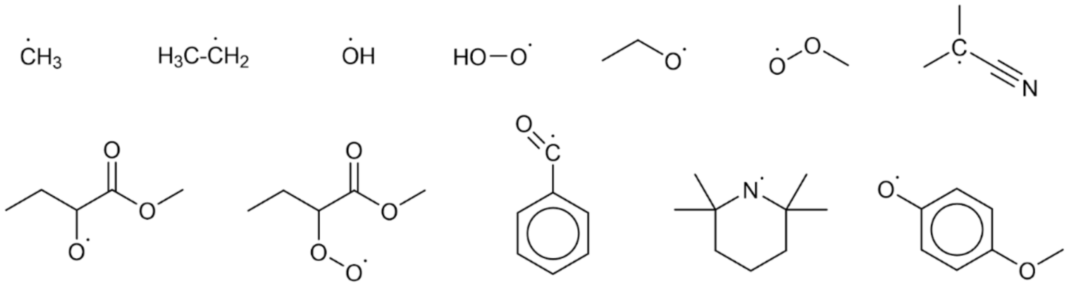

Figure S2. A set of radicals used in training data generation that react with the list of drug-like molecules to generate further radical species.

The test dataset from Ref.[3] centers around main and side reactions relevant to radical polymerization of vinyl ($H_2C{=}CH{-}R$) and methyl-substituted vinyl ($H_2C{=}C(CH_3){-}R$) monomers. The following types of species were considered:

- initial radicals either generated in situ from technically important radical polymerization initiators or as intermediates in the autoxidation chemistry of hydrocarbons,
- a variety of vinyl and methyl-substituted vinyl monomers prone to radical polymerization,
- 2-Mercaptoethanol as a classical chain-transfer agent (CTA),
- propagating radicals derived from the respective monomers,
- and polymer repeating units derived from the respective monomers.

The propagating radicals are model species for the growing polymer chain end radicals. In this dataset, they are derived from the monomers by adding a CH_3 radical to either the terminal or central carbon atom of the C=C double bond, as shown in the top reaction of Figure S3. CTAs are species in which a simple transfer of the radical center from the propagating radicals is possible, usually by a hydrogen abstraction reaction, and in which the radical center formed subsequently reacts with a monomer to start a new propagating chain. Chain transfer reactions are usually categorized by the type of molecule that reacts with the growing chain. In this dataset, the transfer to a repeating polymer unit is considered, represented by the addition of a CH_3 to both sites of the C=C double bond, as exemplified in the bottom reaction of Figure S3.

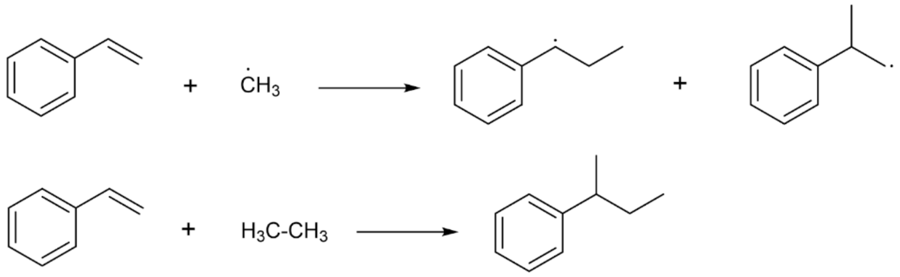

Figure S3. Top: An example of how propagating radicals are formed from a vinyl monomer. Bottom: Example to demonstrate the polymer repeating unit model generation from the corresponding (vinyl) monomer.

The following types of bimolecular reactions of radicals with closed-shell molecules were computed,

- Initial radical + monomer,
- Propagating radical + monomer,
- Initial radical + CTA,
- Propagating radicals + CTA,
- Initial radical + polymer repeating unit,
- Propagating radicals + polymer repeating unit.

All minima and saddle point structures were optimized with TURBOMOLE 7.7.1[7,8] using the functional B3LYP[9,10] with D3 dispersion corrections[11] and Becke-Johnson damping[12] along with a triple ζ polarized split-valence basis set, B3LYP-D3(BJ)/def2-TZVP. The m4 integration grid was used together with tight convergence settings: energy 8, gcart 4, scfconv 8, and denconv $\mathbf{10^{-8}}$. For saddle-point optimizations, the eigenvector-following algorithm as implemented in Turbomole was used. For each reactant, transition state (TS), and product structure, conformational searches were performed using a BASF internal tool that iteratively scans dihedral angles around all rotatable bonds. The generated set of conformers was subsequently optimized using the metaGGA functional TPSS/def2-TZVP[13] and the COSMO($\varepsilon = \infty$) continuum solvation model[14] (with COSMOtherm version 18[15]). From there on, only the lowest energy conformer was reoptimized in the gas phase, at the B3LYP-D3(BJ)/def2-TZVP level of theory with the same settings as the preoptimization procedure described above. Harmonic vibrational frequencies were calculated based on second-order analytical derivatives, verifying that the optimized minima do not contain an imaginary vibrational frequency and that refined transition states contain exactly one imaginary mode. All TS geometries were further verified by a forward and reverse IRC calculation, linking the saddle point to its adjacent reactant and product potential energy surface minima by a mass-weighted downhill optimization. All IRCs were performed with Gaussian 16[16] in gas-phase using the same level of DFT as was used during optimization.

b) BASChem19

Initial structure estimates for each TS was obtained with the molecular growing string method[2,17] and optimized with TPSS-D3/def2-TZVP[13]. For every TS, analytical second derivatives were computed to verify the first-order saddle points. Full relaxation at the same level of theory of each of the 19 transition states then led to the nearest minimum on the potential energy surface, which is either the reactant or product of the respective reaction. In cases where the calculation identified reactant and product conformers that were different than the initial search structures, the IRC structures were taken as reference. The difference in electronic gas phase energy between TS and minima is regarded as the activation barrier. To find the lowest energy structure for TS and minima structures, a conformer search with the CREST algorithm[18] at the GFN2-xTB level of theory was further performed. The reaction coordinate was constrained to retain the TS structure.

All DFT calculations for the BASChem19 benchmark set were performed with TURBOMOLE 7.7.1. Geometries and harmonic vibrational frequencies of stable species and 1st-order saddle points (TS) were obtained using DFT at the TPSS[13]/def2-TZVP level with grid m4, default convergence criteria, and D3-BJ dispersion correction. The harmonic vibrational frequency acknowledges using AOFORCE. Reference single-point energies in the gas phase were calculated with the range-separated hybrid-(meta-)GGA functional ωB97M-D3(BJ)/def2-TZVPP[11,12,19], ωB97X-D/def2-QZVP[20], which includes an empirical dispersion correction, as well as B3LYP-D3/def2-QZVP. To screen for different conformers, the iMTD algorithm implemented in CREST[18] is employed at the GFN2-xTB level of theory. All semiempirical calculations were performed with xtb version 6.7[4,5,21]. All optimizations and single-point energy calculations employed the resolution-of-identity (RI) approximation for the Coulomb integrals using matching default auxiliary basis sets.

c) Radical Polymerization Reaction Case Studies

The benchmark DFT calculations for radical polymerization reaction corresponding to Figures 4, 5 and 6 are performed using the ORCA quantum chemistry software, version 5.0.4[22], with ωB97M range-separated meta-GGA hybrid functional with added D3 (BJ) dispersion correction and def2-TZVPP basis set with def2/J auxiliary basis and the RIJCOSX approximation for Coulomb and exchange integrals. A tight self-consistent field criterion (TIGHTSCF) was used with a tolerance set to $\mathbf{1\,x\,10^{-8}}$ Ha for energy change between two cycles and $\mathbf{5\,x\,10^{-9}}$ root mean square (RMS) density change. Fully converged SCF and stability are enforced with SCFConvForced and slowconv. Geometry optimization was carried out with the TIGHTOPT setting with energy tolerance $\mathbf{1\,x\,10^{-6}}$ Ha, RMS gradient norm tolerance $\mathbf{3\,x\,10^{-5}}$ Ha/Bohr, and RMS displacement tolerance $\mathbf{6\,x\,10^{-4}}$ Bohr. All other parameters were set to default values.

For radical decomposition reactions, a relaxed potential energy scan was carried out across the stretching of the selected bond separately for different spin states (S=1, 3). The number of steps considered along the bond coordinate varied from 15 (Benzopinacol) to 27 (benzoyl peroxide) based on the cost and bond length considered. The Climbing Image Nudged Elastic Band (CI-NEB)[6] method is used to find an approximate TS for the ring-opening polymerization reaction. The maximum number of nodes for this example was set to 25. The highest energy structure from the minimum energy pathway search was used as the starting point for TS optimization, which was later optimized for IRC calculations with the same convergence criteria mentioned above.

The two-dimensional potential energy surface scan for Azobisisobutyronitrile (AIBN) was also carried out using the ORCA package, albeit with B3LYP-D3(BJ)/def2-TZVP level of theory. While the SCF convergence criteria remain unchanged, the geometry optimization was performed with lower precision, with energy tolerance $\mathbf{5\,x\,10^{-6}}$ Ha, RMS gradient norm tolerance $\mathbf{1\,x\,10^{-4}}$ Ha/Bohr, and RMS displacement tolerance $\mathbf{2\,x\,10^{-3}}$ Bohr. Due to computational cost, the 2D scan was performed in four equal parts, with each C-N bond stretching from 1.4 to 2.7 Å and from 2.7 to 3.9 Å, respectively. Each of the four parts considered 81 intermediate geometries for both singlet and triplet scans. Single point energies were then calculated for all geometries with ωB97M-D3(BJ)/def2-TZVPP and with the same convergence criteria as for the rest of the test sets.

Avogadro (version 1.100.0)[23] and UCSF ChimeraX[24] were used for molecular visualization and graphical analysis of molecular structures for all examples.

2. Training Protocol

AIMNet2-NSE models were trained using a structured workflow, repeated consistently across four independent training runs to construct an ensemble model. Each ensemble member followed an identical procedure with controlled random initialization to ensure robust predictive performance.

The AIMNet2 architecture employs a message-passing neural network with charge equilibration integrated into the message-passing iterations. The network performs multiple iterations, controlled by the number of multi-layer perceptrons (MLPs), where atomic features and charges are iteratively refined.
In the first message-passing iteration, the network processes the following,

1. Atomic features (a): Element-specific learned embeddings initialized via the atomic feature vector embedding layer. Each element has a trainable feature vector that the network learns during training.
2. Atomic environment vectors (AEV): Scalar and vector representations of each atom's local chemical environment. These encode radial distances and angular information about neighboring atoms.
3. Neighbor convolution: Information from neighboring atoms is aggregated through convolution operations, allowing each atom to see its chemical environment.

The first MLP processes these combined inputs and produces an output that is then split into three components:

```
_q, _f, delta_a = x.split([
   self.num_charge_channels,
   self.num_charge_channels,
   x.shape[-1] - 2 * self.num_charge_channels, ], dim=-1)
```

Where, _q are Initial atomic charges (1 channel for closed-shell, 2 channels for NSE open-shell), _f is used in subsequent charge equilibration (see Eq. 2 in manuscript), and delta_a are the updates to atomic features for the next iteration. The network output dimension is explicitly set to num_charge_channels, and these specific dimensions are designated to represent charges.

Training of each model employs a composite multi-task loss function ($\boldsymbol{\mathcal{L}}$), which integrates predictions of energies, atomic forces, charges, and spin charges. The loss function consists of a weighted combination of mean squared errors for total energy ($\boldsymbol{\mathcal{L}_E}$), atomic forces ($\boldsymbol{\mathcal{L}_F}$), atomic charges ($\boldsymbol{\mathcal{L}_q}$), and atomic spin charges ($\boldsymbol{\mathcal{L}_s}$),

$$\boldsymbol{\mathcal{L} = w_E\mathcal{L}_E + w_F\mathcal{L}_F + w_q\mathcal{L}_q + w_s\mathcal{L}_s}$$

Weights, $\boldsymbol{w}$, were selected via an empirically guided hyperparameter search. The weights applied are $\boldsymbol{w_E = 0.625\ eV^{-2}}$, $\boldsymbol{w_F = 0.125\ A^2eV^{-2}}$, $\boldsymbol{w_q = 0.125\ e^{-2}}$ and $\boldsymbol{w_s = 0.125\ e^{-2}}$ respectively.

Reference atomic charges and spin charges for open-shell systems are calculated using Hirshfeld population analysis of the spin-polarized electron density obtained from unrestricted DFT calculations with ωB97M-D3(BJ)/def2-TZVPP. During each message-passing iteration, the model first produces initial atomic spin-charge estimates ($\boldsymbol{\widetilde{q_i^\alpha}}$ and $\boldsymbol{\widetilde{q_i^\beta}}$), which are then equilibrated through the NSE block (Equation 2) to ensure charge conservation. The equilibrated charges are fed into subsequent message-passing steps and later used in computing Coulombic contributions to molecular energy. After all iterations, the final predicted spin charges are used to compute total atomic charges ($q_i = q_i^\alpha + q_i^\beta$) and atomic spin charges ($q_i = q_i^\alpha - q_i^\beta$), which are then compared against reference Hirshfeld charges in the loss terms $\mathcal{L}_q$ and $\mathcal{L}_s$.

Optimization was performed using the Rectified Adam (RAdam)**[25]** optimizer with a learning rate initialized at $\boldsymbol{4 \times 10^{-4}}$ and weight decay of $\boldsymbol{1 \times 10^{-8}}$. With a reduced-on-plateau learning rate schedule, training converges within 1.5M steps. To improve training performance, all minibatches were composed of molecules with the same number of atoms to avoid padding operations. Proper data feed shuffling was achieved within the multi-GPU distributed data-parallel (DDP) approach with 4 NVDIA V100 GPUs and a batch size of 150 molecules per GPU. 5% of the training data set was set aside for validation. Self-atomic energies (SAE) were subtracted from the predicted energies during training and inference, using linear regression-fitted values specific to atomic species present in the dataset.

AIMNet2-NSE outputs molecular energy, atomic forces, partial charges, and partial spin charges. To account for non-local dispersion interactions, we incorporated an explicit dispersion correction using a PyTorch implementation of the DFT-D3 model developed by Grimme and coworkers[11,12]. The code, models, and scripts are available at https://github.com/isayevlab/aimnetcentral.

3. AIMNet2-NSE Geometry Optimization

All AIMNet2-NSE geometry optimizations, relaxed potential energy surface (PES) scans, and IRC calculations were performed with our locally developed plugin to the Pysisyphus[26] software. Plugins are available at https://github.com/isayevlab/aimnetcentral. For relaxed bond scan, the number of nodes is matched with the corresponding DFT calculations. AIMNet2-NSE provides analytic gradients obtained through automatic differentiation of the energy with respect to atomic coordinates.This ensures smooth and continuous derivatives suitable for geometry and transition-state optimizations. The tight convergence criteria were applied, where geometry optimizations use a quasi-Newton algorithm with BFGS or L-BFGS Hessian updates[27]. The initial Hessian was computed numerically from model-derived gradients and updated every 10 macro cycles. Optimization thresholds corresponded to Gaussian defaults (thresh: gau), with $\mathbf{4.5\,x\,10^{-4}}$ Ha/Bohr max force, $\mathbf{3.0\,x\,10^{-4}}$ Ha/Bohr RMS force, $\mathbf{1.8\,x\,10^{-3}}$ Bohr max step, and $\mathbf{1.2\,x\,10^{-3}}$ Bohr RMS step. Trust radii were adaptively adjusted between 0.001 and 0.01 Å. For IRC calculations, the default string method is used for reaction path search. Pre-optimization of the reaction path was done using the graph-based search method, with climbing image enabled. The approximate transition-state geometry is refined with the restricted step internal coordinate rational function optimization (RS-I-RFO) approach[28,29] with Hessian updates. IRC propagation was carried out using the Euler Predictor Corrector method[30] implemented in Pysisyphus and the default mass-weighted internal coordinate projection with RMS gradient convergence threshold set at 0.0005 Ha/Bohr.

4. Geometry-Based Similarity and Scaffold Analysis

To ensure that no identical or near-identical configurations appear in both the training and test datasets discussed in Figure 2, we performed a geometry-based data-leakage assessment using three-dimensional (3D) structural similarity metrics. Since both datasets contain open-shell radicals and transition-state geometries where bond connectivity changes continuously along the reaction path, canonical SMILES strings and graph-based molecular descriptors are ill-defined or unstable. For such systems, even geometrically distinct configurations may share the same SMILES representation, leading to false positives or missed duplicates in conventional SMILES-based Tanimoto or Murcko scaffold analyses.

Geometry-Based RMSD Analysis: Each structure (reactant, intermediate, or product) in the test and training datasets was represented by an element-resolved histogram of heavy-atom pairwise distances, providing a 3D fingerprint invariant to translation, rotation, or atom ordering. Reactions were compared at the path level, meaning that all structures along the reaction path (reactants, products, and all intermediate geometries) were considered when searching for matches. For every test reaction, we computed the minimum RMSD between any structure in its path and all structures in the training data. If the minimum RMSD was ≤ 0.05 Å, the reaction was flagged as a duplicate. This stringent geometric threshold ensures that only nearly identical atomic configurations, not merely species with the same formula, are identified as overlapping.

This analysis revealed that only 79 of 4966 test reactions (1.57%) had a geometrically similar configuration within the training data under the RMSD ≤ 0.05 Å threshold. These correspond to near-identical geometries with Tanimoto > 0.6, confirming consistency between the two metrics. If the RMSD tolerance is relaxed to 0.10 Å, the overlap increases to 291 reactions (5.86%). The test set contained 496 unique atomic-number sequences, compared to 9134 unique compositions in the training data.

Geometry-based scaffold analysis: To complement the similarity analysis, we also performed a 3D scaffold analysis tailored for transition-state and radical geometries. Instead of conventional 2D Murcko scaffolds, each structure was converted into a 3D connectivity graph, where heavy atoms within 1.8 Å were treated as connected. The largest connected heavy-atom subgraph defines a geometric scaffold, corresponding to the actual bonded framework present in the geometry.

Using this definition, we identified 173 unique geometric scaffolds in the test set and 2384 in the training data. All 173 test scaffolds were also present in the training set. This is expected, as the training data systematically enumerate all fundamental bonding motifs (C-C, C=C, C-O, C=O, C-S, C-N, etc.) arising from hydrogen-abstraction and radical-addition reactions of small organic molecules. The test set is composed of vinyl, methacrylate, and chain-transfer frameworks that are combinatorial assemblies of these same atomic skeletons. Thus, while the test set shares the same elemental scaffold space, it represents distinct geometries and reaction contexts, confirming that AIMNet2-NSE is evaluated within its intended chemical domain without any leakage of identical configurations.

5. Size Consistency Analysis for Radical Polymerization Reactions

A critical requirement for computational methods intended for polymer chemistry applications is size consistency. The propagation step (addition of a monomer to a growing polymer chain) should have consistent activation and reaction energies independent of chain length, since actual chemistry occurs locally at the radical center.

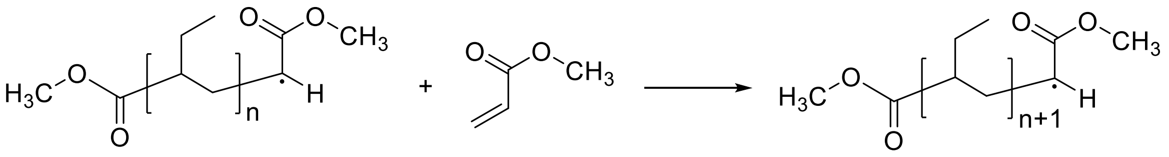

Figure S4. The grwoing-chain plymerization of methyl acrylate

To evaluate whether AIMNet2-NSE maintains size consistency, we performed a systematic study of the radical propagation reaction for methyl acrylate polymerization (Figure S4). We consider sequential addition of five monomeric units, with the largest polymer comprising of 64 atoms. Since calculating reference DFT energies with ωB97M-D3(BJ)/def2-TZVPP for such large systems is prohibitively expensive, we use TPSS/def2-TZVP as our reference method. Calculations were performed in TURBOMOLE, following the same procedure mentioned in Section 1.

TS optimization with AIMNet2-NSE was also performed following the procedure highlighted in Section 1. Activation energy for each polymerization reaction was calculated as *E(TS)-E(Reactant),* and reaction energies are calculated as *E(Product)-E(Reactant)*. Figure S5 shows size consistency analysis for methyl acrylate radical propagation. Individual ensemble models show fluctuations of ±1-3 kcal/mol across chain lengths, which exceeds the strict <0.5 kJ/mol (~0.12 kcal/mol) criterion typically applied to DFT size consistency tests. However, this variance is inherent to ML models and represents epistemic uncertainty rather than systematic error. The averages depicted in Table S1(a) and Table S1(b) show better consistency, and the variance does not grow with system size, indicating stable predictions.

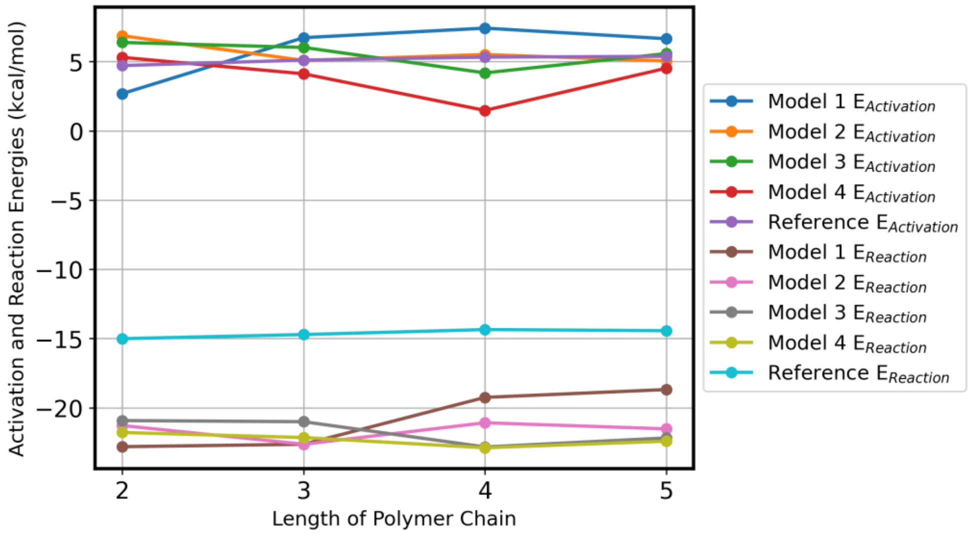

Figure S5. Size consistency analysis for methyl acrylate radical polymerization. Activation energies ($E_{Activation}$, upper cluster) and reaction energies ($E_{Reaction}$, lower cluster) are shown as a function of polymer chain length (n = 2-5 monomer units). All four AIMNet2-NSE models (Model 1-4) are compared with TPSS/def2-TZVP reference calculations. Size consistency would be indicated by horizontal lines (constant energy regardless of chain length).

Table S1. (a) Activation energies (kcal/mol) for methyl acrylate radical polymerization as a function of chain length, (b) Reaction energies (kcal/mol) for methyl acrylate radical polymerization as a function of chain length.

**Chain Length (n)**	**Model 1 $\Delta E^{\ddagger}$**	**Model 2 $\Delta E^{\ddagger}$**	**Model 3 $\Delta E^{\ddagger}$**	**Model 4 $\Delta E^{\ddagger}$**	**Avg $\Delta E^{\ddagger}$**	**Std Dev**	**TPSS/def2-TZVP $\Delta E^{\ddagger}$**
**2**	2.7	6.9	6.4	5.3	5.3	1.9	4.7
**3**	6.7	5.1	6.0	4.1	5.5	1.2	5.1
**4**	7.4	5.5	4.2	1.5	4.6	2.5	5.3
**5**	6.7	5.0	5.6	4.5	5.5	0.9	5.4
**Chain Length (n)**	**Model 1 ΔE**	**Model 2 ΔE**	**Model 3 ΔE**	**Model 4 ΔE**	**Avg ΔE**	**Std Dev**	**TPSS/def2-TZVP ΔE**
**2**	-22.8	-21.3	-20.9	-21.8	-21.7	0.9	-15.0
**3**	-22.6	-22.6	-21.0	-22.2	-22.1	0.7	-14.7
**4**	-19.3	-21.1	-22.8	-22.9	-21.5	1.8	-14.4
**5**	-18.7	-21.5	-22.2	-22.4	-21.2	1.7	-14.4

The size consistency observed here demonstrates that AIMNet2-NSE's neural spin-charge equilibration correctly captures the local electronic structure around the radical center. The fact that energies remain stable even though training data primarily consisted of smaller molecules (typically <50 heavy atoms) validates that the model has learned physically meaningful representations rather than memorizing specific molecular sizes.

While there is a ~7 kcal/mol difference between AIMNet2-NSE and TPSS reaction energies as AIMNet2-NSE is trained on a different level of theory, for screening applications and relative energy predictions, which constitute the majority of computational polymer chemistry workflows, size consistency is more critical than absolute accuracy. AIMNet2-NSE demonstrates robust size consistency for radical polymerization reactions, with activation and reaction energies remaining stable across variable polymer chain lengths.

6. Additional Benchmarks and Results

a) GMTKN55[31] and NCI[32] Atlas

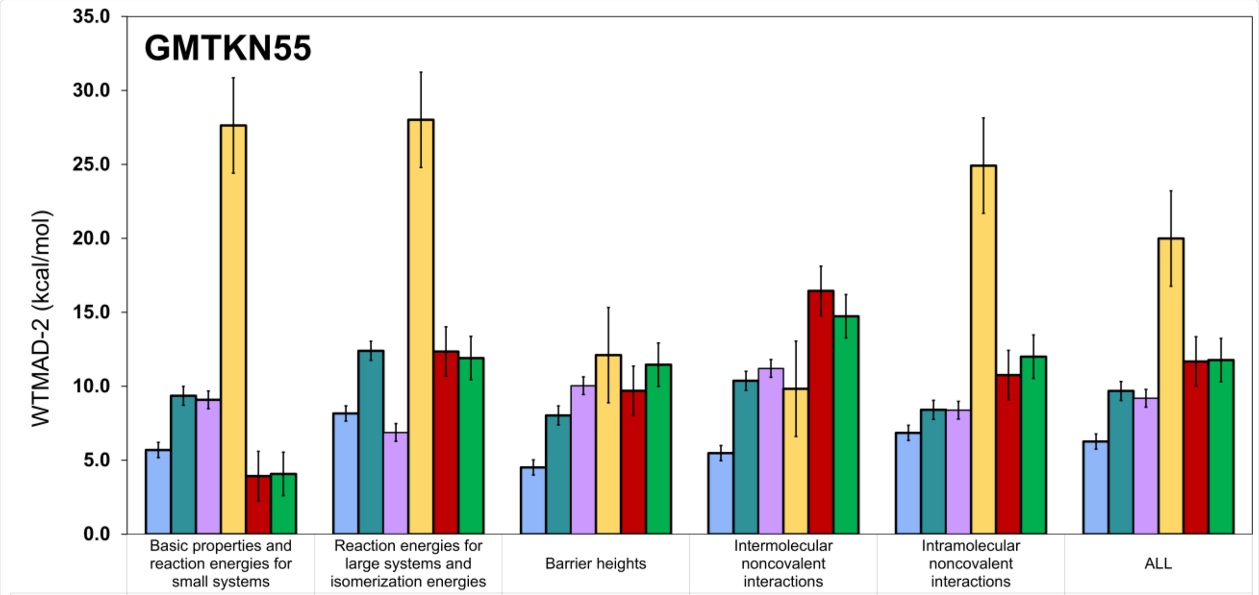

	Basic properties and reaction energies for small systems	Reaction energies for large systems and isomerization energies	Barrier heights	Intermolecular noncovalent interactions	Intramolecular noncovalent interactions	ALL
ωB97X-D3	5.69	8.16	4.51	5.48	6.85	6.27
B97-3c	9.36	12.40	8.03	10.37	8.41	9.68
OrbNet Denali	9.08	6.87	10.04	11.20	8.38	9.19
GFN2-xTB	27.63	28.02	12.11	9.83	24.92	19.99
AIMNet2	3.93	12.35	9.69	16.45	10.76	11.68
AIMNet2-NSE	4.07	11.91	11.46	14.74	12.00	11.77

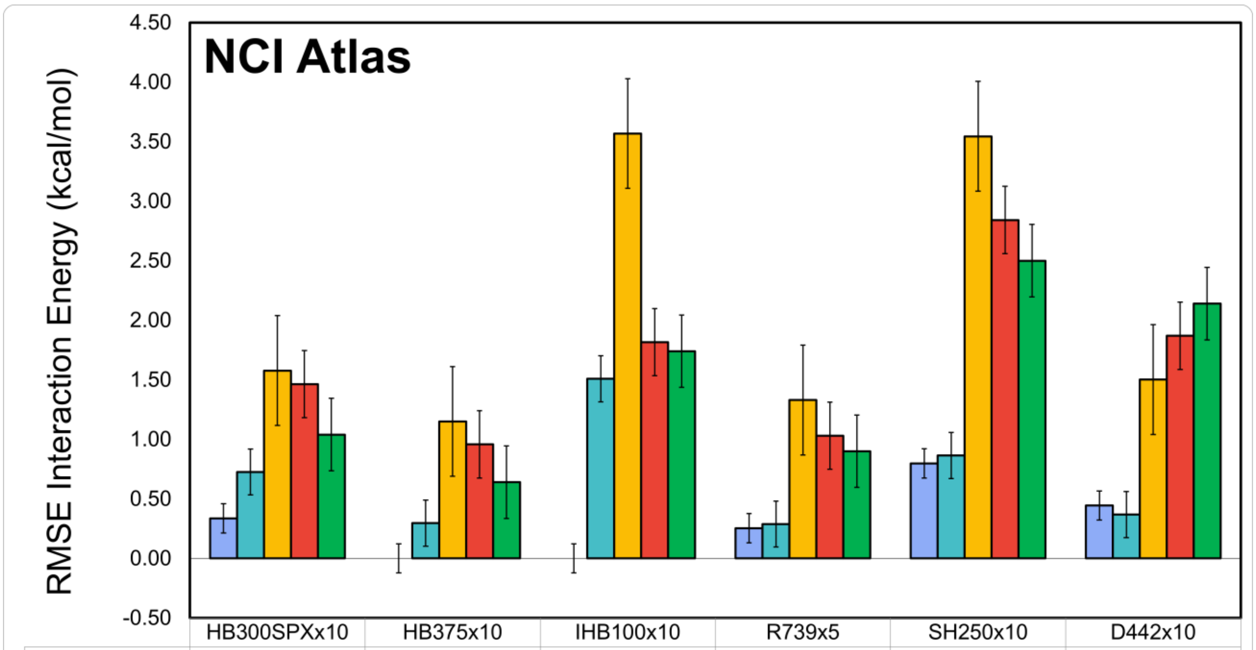

	HB300SPXx10	HB375x10	IHB100x10	R739x5	SH250x10	D442x10
wB97X-V	0.34			0.25	0.80	0.44
B3LYP-D3(BJ)	0.73	0.30	1.51	0.29	0.86	0.37
GFN2-XTB	1.58	1.15	3.57	1.33	3.54	1.50
AIMNet2	1.46	0.96	1.82	1.03	2.84	1.87
AIMNet2-NSE	1.04	0.64	1.74	0.9	2.5	2.14

Figure S6. Performance of several DFT hybrid and GGA functionals, GFN2-xTB, AIMNet2, and AIMNet2-NSE on the GMNTK55 benchmark (closed-shell, top) and Non-Covalent Interaction (NCI) Atlas benchmark (closed-shell, bottom). HB300SPX×10 - Hydrogen bonding extended to S, P, and halogens; HB375×10 - Hydrogen bonding in organic molecules; IHB100×10 - Ionic hydrogen bonds in organic molecules; R739×5 - Repulsive contacts in an extended chemical space; SH250×10 - Sigma-hole interactions; D442×10 - London dispersion in an extended chemical space.

Table S2. Mean absolute errors (MAE) in energy in kcal/mol for GMTKN55 subsets (closed-shell, charged)

Subset	ωB97X-D3	B97-3c	OrbNet Denali	GFN2-xTB	AIMNet2	AIMNet2NSE
**PA26**	3.32	5.58	5.29	163.05	3.33	3.2
**NBPRC**	1.67	1.56	10.27	10.51	1.5	1.88
**FH51**	2.47	4.42	3.58	11.41	1.49	1.85
**TAUT15**	1.05	1.71	1.13	0.98	0.74	0.64
**DARC**	1.27	4.34	1.31	17.77	1.04	1.15
**BSR36**	4.36	1.90	0.77	2.76	9.1	8.56
**CDIE20**	0.72	1.98	0.61	1.80	0.69	0.78
**ISO34**	1.18	1.87	1.21	6.90	0.76	0.77
**ISOL24**	2.75	5.19	2.64	11.68	2.4	2.36
**C60ISO**	1.18	6.27	11.82	5.80	17.28	9.17
**PArel**	0.67	1.80	1.60	5.86	0.94	1.01
**BHPERI**	2.85	4.59	4.72	10.24	6.61	7.4
**BHDIV10**	1.01	5.80	6.83	8.12	4.44	3.67
**INV24**	1.63	1.96	4.59	3.32	2.9	2.76
**BHROT27**	0.47	0.61	0.39	1.17	0.84	0.78
**PX13**	3.18	7.08	14.84	2.74	7.46	15.5
**WCPT18**	2.14	5.46	4.91	3.84	4.28	4.42
**ADIM6**	0.36	0.53	0.40	1.15	1.48	0.18
**S22**	0.36	0.29	0.45	0.76	0.89	0.6
**S66**	0.52	0.32	0.48	0.73	0.63	0.48
**WATER27**	14.23	9.41	2.39	3.05	8.68	7.9
**CARBHB12**	0.83	2.07	0.91	1.79	2.39	1.83
**PNICO23**	0.38	1.64	1.71	1.11	3.42	3.82
**HAL59**	0.34	1.62	2.15	1.28	2.52	2.26
**AHB21**	3.40	3.27	1.81	2.97	1.44	1.4
**IL16**	2.09	2.34	4.60	4.32	1.29	1.44
**IDISP**	2.78	3.91	2.61	6.78	1.35	2.67
**ICONF**	0.34	0.38	1.25	1.63	0.66	0.71
**ACONF**	0.09	0.21	0.06	0.19	0.17	0.24
**Amino20x4**	0.26	0.33	0.35	0.95	0.54	0.46
**PCONF21**	0.33	0.83	0.47	1.76	1.01	0.86
**MCONF**	0.48	0.33	0.42	1.72	0.43	0.72
**SCONF**	0.30	0.77	0.32	1.64	1.3	0.79
**UPU23**	0.94	0.51	0.87	2.91	2.02	2.29
**BUT14DIOL**	0.41	0.41	0.4	1.25	0.15	0.46

b) Ionization potentials and electron affinity benchmarks

We evaluated AIMNet2-NSE for the G21IP (ionization potentials) and G21EA (electron affinity) subsets of GMTKN55 benchmark database. Both datasets contain small organic molecules, and the benchmarks are calculated against back-corrected experimental reference values. We also include ωB97X-D3(0)/def2-QZVP results for comparison[31]. G21IP benchmarks are presented in Table S3, and G21EA benchmarks are presented in Table S4. Performance analysis reveals that prediction accuracy varies systematically across molecular types, with triple-bonded diatomic molecules presenting the greatest challenge. This is consistent with the underrepresentation of such highly unsaturated small molecules in our training dataset, highlighting opportunities for targeted data augmentation in future model iterations.

Table S3. Deviations in kcal/mol in ionization potential predictions calculated with ωB97X-D3 and AIMNet2-NSE for molecules in the G21IP dataset

	Cation	Neutral	Reference	ωB97X-D3 (Δ)	AIMNet2-NSE (Δ)
1	CH_4^+	CH_4	296.339	-4.1	4.43
2	NH_3^+	NH_3	235.69	-1.75	1.81
3	OH^+	OH	300.917	1.21	29
4	H_2O^+	H_2O	292.648	-2.72	-1.27
5	HF^+	HF	371.311	-2.52	-1.42
6	SiH_4^+	SiH_4	255.387	-0.76	3.31
7	PH^+	PH	234.107	-0.06	4.24
8	PH_2^+	PH_2	226.367	1.24	0.19
9	PH_3^+	PH_3	227.822	-2.22	4.12
10	SH^+	SH	239.3	1.12	9.17
11	HCl^+	HCl	294.459	-0.49	0.19
12	$C_2H_2^+$	C_2H_2	264.585	-6.72	-5.13
13	$C_2H_4^+$	C_2H_4	243.709	-6.91	-4.64
14	CO^+	CO	322.986	2.16	10.04
15	N_2^+	N_2	359.365	6.91	11.45
16	O_2^+	O_2	277.727	5.9	6.84
17	P_2^+	P_2	242.854	-3.42	-0.32
18	S_2^+	S_2	215.737	4.54	1.96
19	Cl_2^+	Cl_2	265.083	-1.75	-1.42
20	ClF^+	ClF	291.699	-2.69	-0.67
21	CS^+	CS	261.153	1.3	16.33
MAE				2.88	5.62

Table S4. Deviations in kcal/mol in electron affinity predictions calculated with ωB97X-D3 and AIMNet2-NSE for molecules in the G21EA dataset

	Neutral	Anion	Reference	**ωB97X-D3 (Δ)**	**AIMNet2-NSE (Δ)**
**1**	CH	CH^-	27.9	0.36	-22.94
**2**	CH_2	CH_2^-	13.4	-0.44	-4.88
**3**	CH_3	CH_3^-	1.2	-2.14	-2.84
**4**	NH	NH^-	8.3	-0.95	-8.3
**5**	NH_2	NH_2^-	16.8	-2.08	-9.42
**6**	OH	OH^-	41.7	-2.36	-12.63
**7**	SiH	SiH^-	29.3	-1.59	-30.43
**8**	SiH_2	SiH_2^-	25.1	-0.14	-8.02
**9**	SiH_3	SiH_3^-	31.4	-0.83	-2.5
**10**	PH	PH^-	23.5	0.52	-8.7
**11**	PH_2	PH_2^-	28.8	-0.71	-4.11
**12**	SH	SH^-	54.2	-1.58	-5.27
**13**	O_2	O_2^-	9.5	-3.67	-7.19
**14**	NO	NO^-	-0.2	3.13	-6.88
**15**	CN	CN^-	89.5	4.46	4.16
**16**	PO	PO^-	24.9	1.46	-9.34
**17**	S_2	S_2^-	38	-0.81	-2.68
**18**	Cl	Cl^-	54.7	3.15	-0.38
**MAE**				1.69	8.37

c) Evaluation of atomic and spin charges:

Figure S7(a) shows the Hirshfeld atomic charges obtained from ωB97M-D3(BJ) and the AIMNet2-NSE predicted charges for benzene. Both methods correctly predict a neutral molecule. The AIMNet2-NSE computed spin charges are 0.000135, 0.000137, 0.000140, 0.000136, 0.000140, −0.000137, −0.000137, −0.000138, −0.000137, −0.000136, and −0.000138 (positive on carbon and negative on hydrogen), summing exactly to zero for this closed-shell system.

Figure S7(b) presents dibenzo[a,f]pentalene, a ground-state open-shell singlet molecule. This example highlights a class of systems where AIMNet2-NSE cannot accurately predict spin-charge distributions. Since the model only receives total molecular charge and spin multiplicity as inputs, it cannot distinguish between closed-shell and open-shell singlet configurations that share the same overall multiplicity. Our current training data does not contain any broken-symmetry open-shell singlet solutions. In a standard unrestricted DFT calculation for a singlet (S=1) without explicit broken-symmetry initialization, the calculation will collapse to a restricted closed-shell solution, yielding

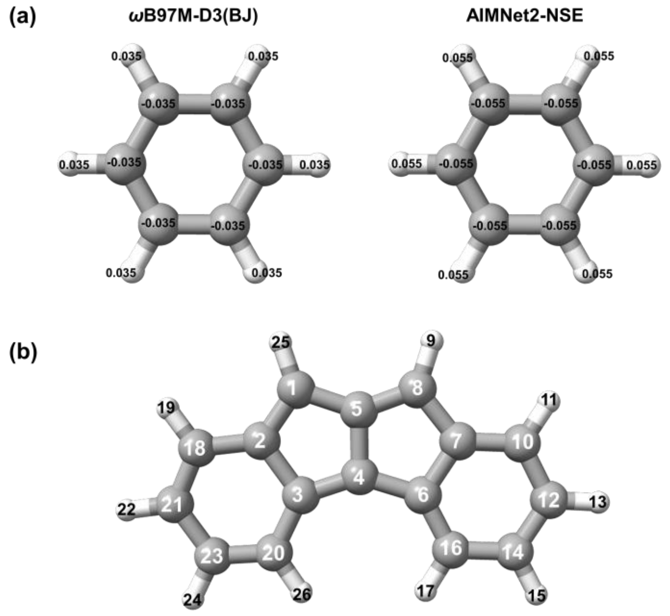

Figure S7. (a) Benzene: Total atomic charges obtained from Hirshfeld population analysis of ωB97M-D3(BJ) electron density compared with AIMNet2-NSE predictions. (b) Dibenzo[a,f]pentalene; a ground-state open-shell singlet molecule.

incorrect charge and spin distributions for systems with diradical characters. Because our training dataset does not include broken-symmetry DFT calculations for open-shell singlets, the model has never been exposed to molecules such as dibenzo[a,f]pentalene and therefore cannot learn their corresponding charge distribution. Integrating the training data with biradicaloid configurations obtained using broken-symmetry DFT, capturing diradical character and singlet-triplet near-degeneracies along bond-breaking coordinates, will directly expose AIMNet2-NSE to open-shell singlet references and improve its ability to describe charge distribution in molecules like dibenzo[a,f]pentalene. The DFT-calculated atomic charges and spin charges for singlet, triplet, and open-shell singlet states of dibenzo[a,f]pentalene are summarized in Table S5, while the corresponding AIMNet2-NSE predictions for singlet and triplet configurations are provided in Table S6.

Table S5. Total atomic charges and spin charges obtained from Hirshfeld population analysis of *ω*B97M-D3(BJ) electron density for dibenzo[a,f]pentalene in singlet, triplet and open-shell singlet spin states

		**Singlet**		**Triplet**		**Open-Shell Singlet**	
	Atom	Charges	Spin Charges	Charges	Spin Charges	Charges	Spin Charges
**1**	C	0.009	0	-0.024	0.404	-0.037	-0.278
**2**	C	-0.010	0	-0.005	0.061	0.000	0.097
**3**	C	0.000	0	-0.014	0.047	-0.012	-0.074
**4**	C	-0.031	0	0.025	0.377	0.017	0.371
**5**	C	-0.021	0	-0.038	-0.002	-0.022	0.095
**6**	C	0.005	0	-0.014	0.047	-0.012	-0.075
**7**	C	-0.049	0	-0.005	0.061	0.000	0.098
**8**	C	-0.049	0	-0.024	0.404	-0.038	-0.279
**9**	H	0.039	0	0.041	0.029	0.039	-0.020
**10**	C	-0.031	0	-0.036	0.056	-0.036	-0.097
**11**	H	0.039	0	0.037	0.003	0.037	-0.007
**12**	C	-0.037	0	-0.032	0.073	-0.030	0.119
**13**	H	0.035	0	0.036	0.005	0.036	0.009
**14**	C	-0.028	0	-0.040	0.053	-0.040	-0.098
**15**	H	0.036	0	0.035	0.004	0.035	-0.008
**16**	C	-0.036	0	-0.028	0.075	-0.028	0.117
**17**	H	0.038	0	0.037	0.004	0.038	0.008
**18**	C	-0.034	0	-0.036	0.056	-0.036	-0.097
**19**	H	0.037	0	0.037	0.003	0.037	-0.007
**20**	C	-0.037	0	-0.028	0.075	-0.028	0.116
**21**	C	-0.042	0	-0.032	0.073	-0.030	0.118
**22**	H	0.034	0	0.036	0.005	0.036	-0.009
**23**	C	-0.033	0	-0.041	0.053	-0.040	-0.098
**24**	H	0.035	0	0.035	0.004	0.035	-0.008
**25**	H	0.050	0	0.041	0.028	0.039	-0.020
**26**	H	0.036	0	0.037	0.004	0.038	0.008
**Total**		0.000	0	0.000	2.000	0.000	0.000

Table S6. Total atomic charges and spin charges predicted with AIMNet2-NSE for dibenzo[a,f]pentalene in singlet and triplet spin states.

		**Singlet**		**Triplet**	
	Atom	AIMNet2-NSE Charges	AIMNet2-NSE Spin Charges	AIMNet2-NSE Charges	AIMNet2-NSE Spin Charges
**1**	C	-0.020	0.002	-0.034	0.372
**2**	C	-0.038	0.000	-0.035	0.062
**3**	C	-0.050	0.001	-0.050	0.069
**4**	C	-0.042	0.011	-0.019	0.282
**5**	C	-0.014	0.012	-0.037	0.105
**6**	C	-0.050	-0.001	-0.050	0.069
**7**	C	-0.036	0.000	-0.035	0.062
**8**	C	-0.035	0.004	-0.034	0.372
**9**	H	0.076	-0.002	0.067	0.019

**10**	C	-0.047	-0.002	-0.034	0.049
**11**	H	0.066	-0.001	0.063	0.016
**12**	C	-0.046	-0.003	-0.046	0.069
**13**	H	0.060	-0.001	0.057	0.018
**14**	C	-0.045	-0.003	-0.046	0.062
**15**	H	0.060	-0.001	0.058	0.018
**16**	C	-0.048	-0.001	-0.037	0.040
**17**	H	0.071	0.000	0.065	0.014
**18**	C	-0.047	-0.003	-0.034	0.049
**19**	H	0.068	-0.001	0.063	0.016
**20**	C	-0.047	-0.001	-0.038	0.040
**21**	C	-0.051	-0.003	-0.046	0.069
**22**	H	0.061	-0.001	0.057	0.018
**23**	C	-0.050	-0.003	-0.046	0.062
**24**	H	0.061	-0.002	0.058	0.018
**25**	H	0.073	-0.001	0.067	0.019
**26**	H	0.070	0.001	0.065	0.014
**Total**		0.000	0.000	0.000	2.000

d) Treatment of dications and quintets

Dications are well-represented in the AIMNet2 training dataset (see Supplementary Figure 3, Anstine et al., Ref. 41), enabling AIMNet2-NSE to handle such systems with ease. We performed geometry optimization and ground-state energy comparisons for Paraquat (a representative dication, Figure S8). Starting from the same input geometry, we optimized structures using both ωB97M-D3(BJ)/def2-TZVPP and AIMNet2-NSE.

AIMNet2-NSE demonstrates reasonable accuracy: atomization energies differ by only 0.87 kcal/mol compared to DFT reference values. The average geometry RMSD across the four AIMNet2-NSE models is 0.06 Å, indicating excellent structural agreement.

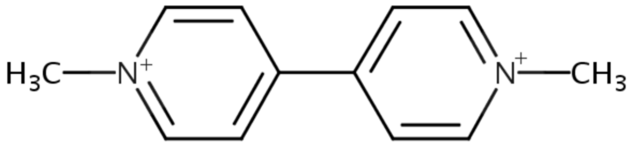

Figure S8. Paraquat or *N*,*N*′-dimethyl-4,4′-bipyridinium dication ($[(C_6H_7N)_2]^{2+}$), also known as methyl viologen. Paraquat is a highly toxic organic compound and is widely used as herbicide.

However, the spin multiplicities represented in the training data were limited to singlet, doublet, and triplet states, reflecting the most commonly encountered cases in organic chemistry. Consequently, AIMNet2-NSE is not expected to generalize reliably to higher-spin states. Figure S9 illustrates the O_2 dissociation energy curve, a challenging example not directly represented in the training data. Since the ground states of both molecular and atomic oxygen are triplets, the dissociation limit corresponds to an overall quintet spin state. As expected, AIMNet2-NSE reproduces the ground-state triplet potential accurately, but the model exhibits larger deviations and higher uncertainty (standard deviation across ensemble members) in the dissociation region. In addition, the high-energy quintet surface of molecular oxygen is poorly approximated, which is consistent with the absence of such high-spin examples in the training data.

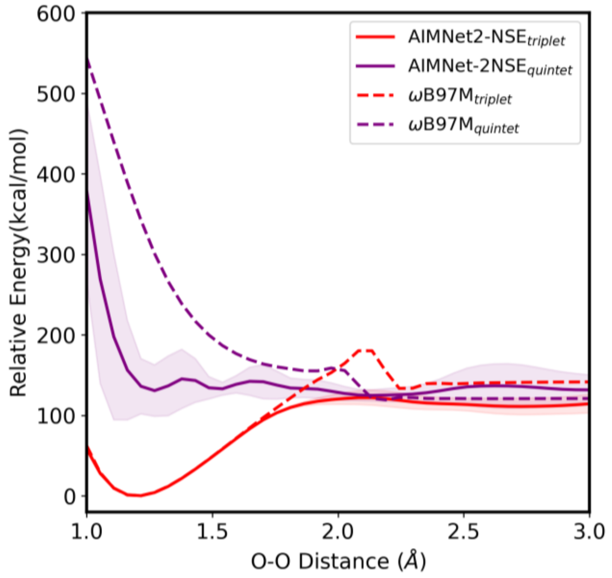

Figure S9. Comparison of AIMNet2-NSE and ωB97M-D3(BJ)/def2-TZVPP energies for the homolytic bond-dissociation of molecular oxygen in both triplet and quintet states

e) Miscellaneous

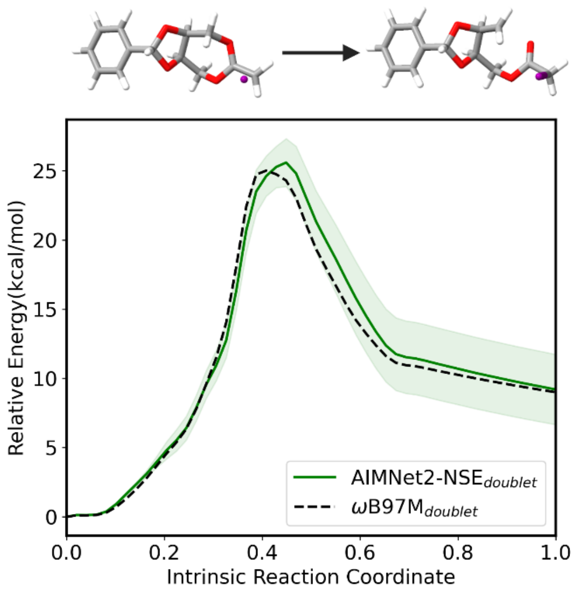

Figure S10. Iodine-mediated ring opening polymerization of 2-methylene-l,3-dioxepane. A comparison of doublet IRCs obtained from AIMNet2-NSE on DFT geometries, and ωB97M-D3(BJ)/def2-TZVPP energies. Trajectories were calculated with an ensemble of four AIMNet2-NSE models, and mean, and standard deviation are depicted here

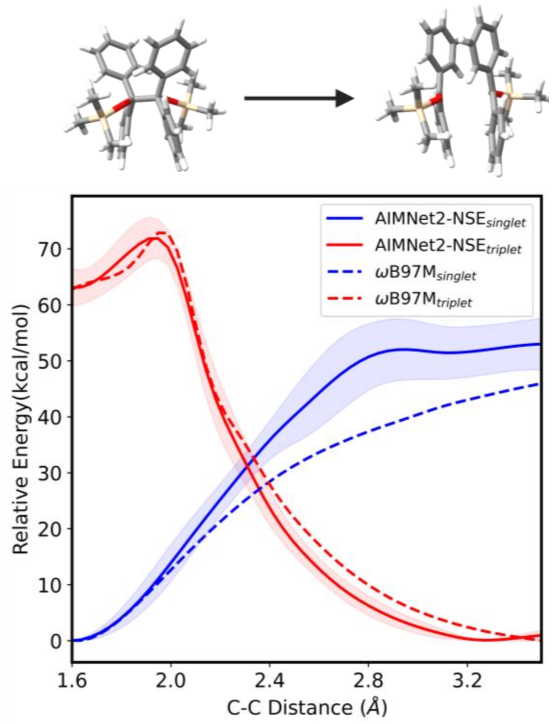

Figure S11. AIMNet2-NSE energies evaluated on the DFT geometries and comparison with corresponding DFT values (ωB97M-D3(BJ)/def2-TZVPP) for the homolytic bond-dissociation of silylated benzopinacol, in both singlet and triplet spin states. Trajectories were calculated with an ensemble of four AIMNet2-NSE models and mean, and standard deviations are depicted here.

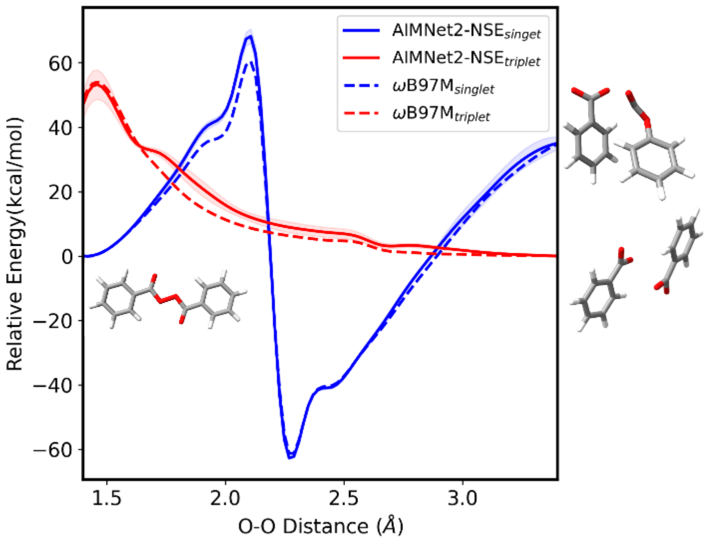

Figure S12. AIMNet2-NSE energies evaluated on the DFT geometries and comparison with corresponding DFT values (*ω*B97M-D3(BJ)/def2-TZVPP) for dissociation of dibenzoyl peroxide, for both spin states. A potential surface energy scan done for the singlet state undergoes rearrangement around 2.1 Å to form two different closed-shell products. Trajectories were calculated with an ensemble of four AIMNet2-NSE models.

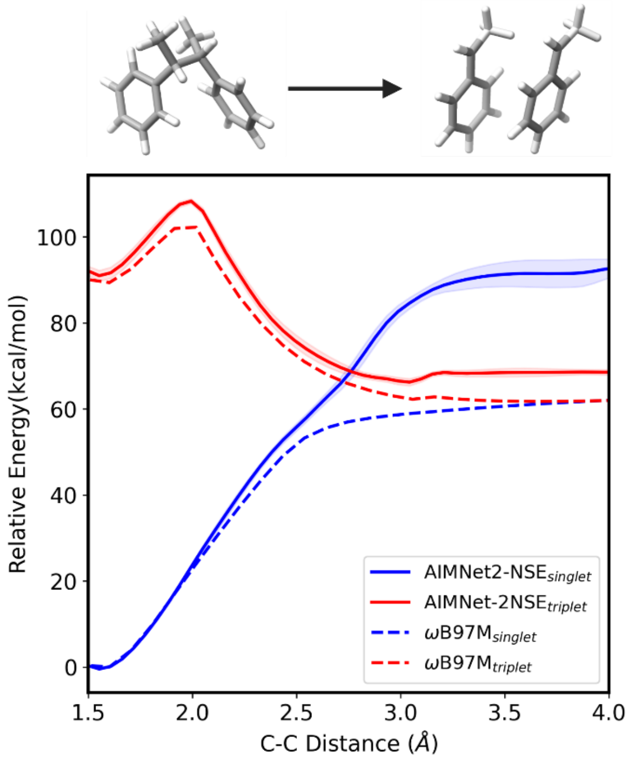

Figure S13. AIMNet2-NSE energies evaluated on the DFT geometries and comparison with corresponding unrestricted DFT values (*ω*B97M-D3(BJ)/def2-TZVPP) for the homolytic bond-dissociation of styrene, for both S=1 and S=3 spin states. Trajectories were calculated with an ensemble of four AIMNet2-NSE models.

Table S7. Comparison between ωB97M-D3(BJ)/def2-TZVPP and AIMNet2-NSE activation barriers and reaction energies obtained from thermochemical analysis for the iodine mediated ring-opening polymerization of 2-methylene-1,3-dioxepane. AIMNet2-NSE predictions are made with a single model instead of an ensemble of four models. All values are in kcal/mol.

Property	Activation Barrier (DFT)	Activation Barrier (AIMNet2-NSE)	Reaction Energy (DFT)	Reaction Energy (AIMNet2-NSE)
Electronic Energy	27.15	29.72	3.45	2.45
Enthalpy (ΔH)	24.76	27.94	1.21	2.45
Gibbs Free Energy (ΔG)	25.27	28.24	−0.41	0.16

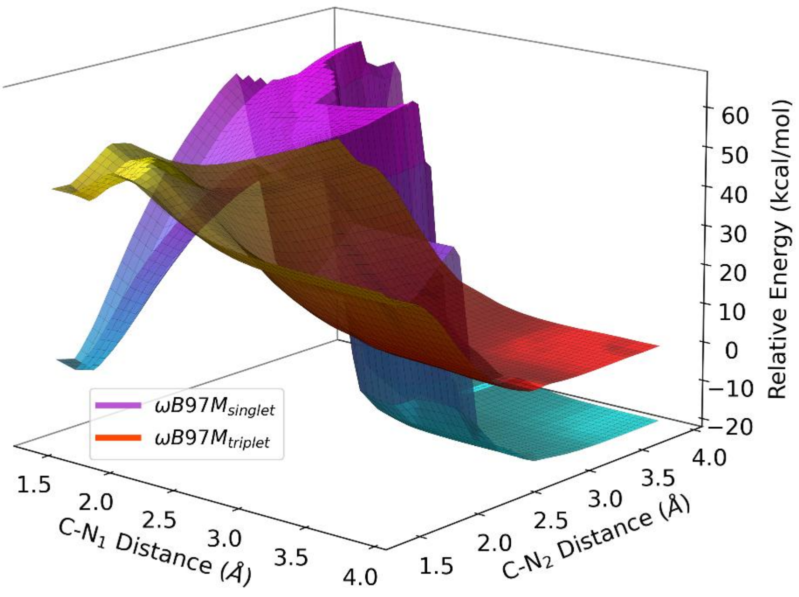

Figure S14. A 2D scan of the Thermal decomposition of AIBN with the dissociation of N_2 as calculated by DFT. Single point energies are calculated with *ω*B97M-D3(BJ)/def2-TZVPP on B3LYP geometries. The AIMNet2-NSE predictions across the two surfaces are shown in Figure 6.

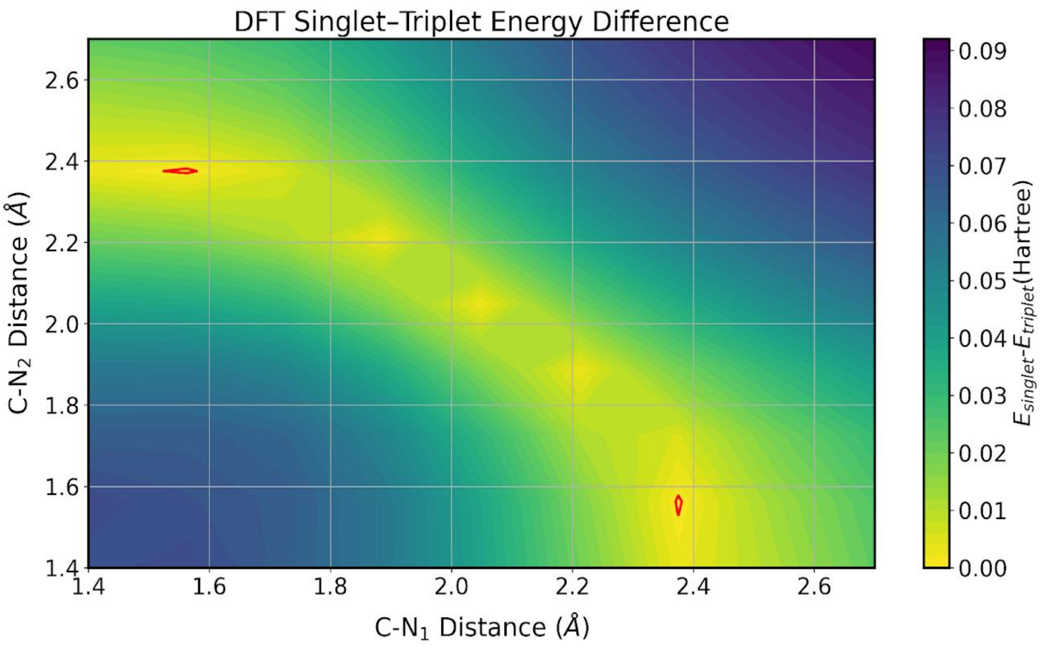

Figure S15. The energy difference between the singlet and triplet surfaces for thermal decomposition of AIBN with the dissociation of N_2 as calculated by DFT. Single point energies are calculated with ωB97M-D3(BJ)/def2-TZVPP on B3LYP geometries.

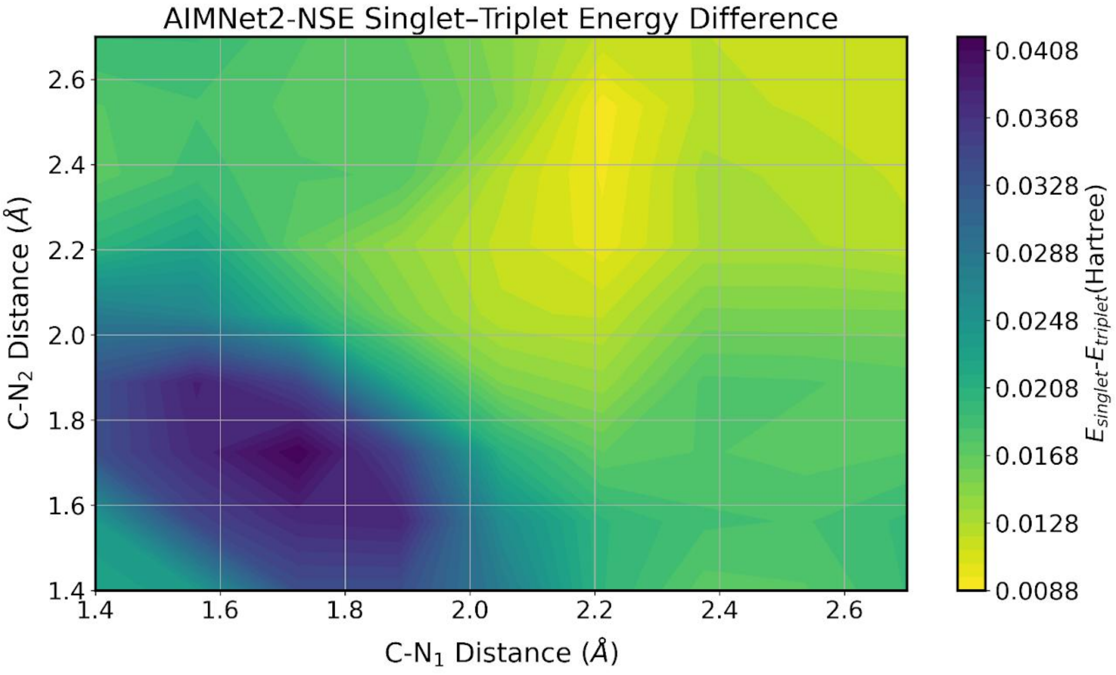

Figure S16. The energy difference between the singlet and triplet surfaces for thermal decomposition of AIBN with the dissociation of N_2 as calculated with AIMNet2-NSE on B3LYP geometries.

Table S8. The final dissociated radical geometries obtained from ωB97M-D3(BJ)/def2-TZVPP and each of the four AIMNet2-NSE models for the dissociation of dibenzoyl peroxide and styrene dimer

	DFT	AIMNet2-NSE1	AIMNet2-NSE2	AIMNet2-NSE3	AIMNet2-NSE4
**Dissociated dibenzoyl peroxide**	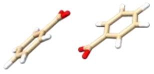	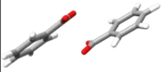	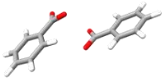	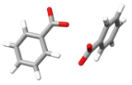	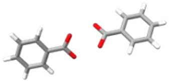
**Dissociation of styrene dimer**	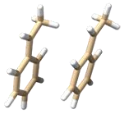	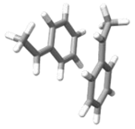	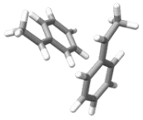	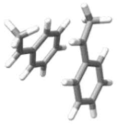	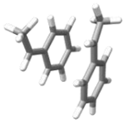

Table S9. Five closest points of contact between the singlet and triplet PES calculated with ωB97M-D3(BJ)/def2-TZVPP for thermal decomposition of AIBN

x	y	E_singlet (Hartree)	E_triplet (Hartree)	|ΔE| (Hartree)
1.5625	2.375	-531.312462	-531.311988	0.00047
2.375	1.5625	-531.312514	-531.311987	0.00053
2.05	2.05	-531.309562	-531.311938	0.00238
2.2125	1.8875	-531.308404	-531.311004	0.00260
1.8875	2.2125	-531.308371	-531.311013	0.00264

Table S10. Five closest points of contact between the singlet and triplet surfaces calculated with AIMNet2-NSE for thermal decomposition of AIBN

x	y	E_singlet (Hartree)	E_triplet (Hartree)	|ΔE| (Hartree)
2.2125	2.5375	-531.329314	-531.338445	0.00913
2.2125	2.375	-531.323493	-531.332950	0.00946
2.2125	2.2125	-531.314514	-531.324388	0.00987
2.5375	2.7	-531.345986	-531.357363	0.01138
2.7	2.7	-531.349993	-531.361371	0.01138

Table S11. Comparison of vibrational frequencies calculated with *ω*B97M-D3(BJ)/def2-TZVPP and AIMNet2-NSE for 2-methylene-1,3-dioxepane. Frequency analysis was performed on the same optimized geometry. AIMNet2-NSE predictions were obtained using a single model rather than an ensemble of four models. All frequencies are in cm^{-1}.

Mode	DFT	AIMNet2-NSE	Δ	% Err	Mode	DFT	AIMNet2-NSE	Δ	% Err
1	18.3	15.8	−2.5	−13.5	46	1071.4	1066.5	−4.9	−0.5
2	34.0	25.1	−8.9	−26.2	47	1090.7	1086.2	−4.4	−0.4
3	48.4	33.3	−15.1	−31.2	48	1111.6	1092.6	−19.0	−1.7
4	57.0	49.5	−7.5	−13.2	49	1114.1	1105.1	−9.0	−0.8
5	78.4	83.3	+4.9	+6.2	50	1145.9	1119.1	−26.8	−2.3
6	85.6	86.9	+1.3	+1.5	51	1176.7	1156.1	−20.6	−1.7
7	153.3	153.4	+0.1	+0.1	52	1177.7	1167.2	−10.5	−0.9
8	170.7	173.7	+2.9	+1.7	53	1180.1	1174.4	−5.7	−0.5

9	175.5	192.8	+17.3	+9.8	54	1203.0	1195.8	−7.2	−0.6
10	202.5	247.1	+44.7	+22.1	55	1250.7	1199.0	−51.7	−4.1
11	252.6	263.8	+11.2	+4.4	56	1267.3	1232.5	−34.8	−2.7
12	267.7	286.3	+18.6	+6.9	57	1290.1	1240.7	−49.4	−3.8
13	318.1	317.2	−0.9	−0.3	58	1305.1	1258.6	−46.5	−3.6
14	328.5	336.1	+7.6	+2.3	59	1316.4	1282.0	−34.4	−2.6
15	375.2	386.9	+11.7	+3.1	60	1331.0	1297.5	−33.5	−2.5
16	393.4	389.9	−3.5	−0.9	61	1347.8	1314.3	−33.5	−2.5
17	422.5	398.6	−23.9	−5.7	62	1357.2	1331.8	−25.4	−1.9
18	430.8	437.9	+7.1	+1.6	63	1378.4	1343.0	−35.3	−2.6
19	478.1	468.0	−10.1	−2.1	64	1383.7	1372.8	−10.9	−0.8
20	523.6	514.2	−9.4	−1.8	65	1390.6	1402.2	+11.5	+0.8
21	628.2	598.1	−30.1	−4.8	66	1406.2	1408.5	+2.2	+0.2
22	632.2	621.7	−10.5	−1.7	67	1412.2	1421.5	+9.3	+0.7
23	660.2	635.1	−25.1	−3.8	68	1433.5	1437.4	+3.9	+0.3
24	670.9	643.8	−27.1	−4.0	69	1436.4	1442.1	+5.7	+0.4
25	724.9	649.0	−75.9	−10.5	70	1485.2	1447.7	−37.5	−2.5
26	729.0	676.4	−52.6	−7.2	71	1497.1	1477.7	−19.4	−1.3
27	752.4	712.7	−39.6	−5.3	72	1507.2	1497.6	−9.6	−0.6

28	765.0	755.8	−9.2	−1.2	73	1544.0	1519.9	−24.1	−1.6
29	773.8	764.0	−9.8	−1.3	74	1654.5	1638.5	−16.0	−1.0
30	800.7	788.9	−11.9	−1.5	75	1664.9	1667.5	+2.6	+0.2
31	820.3	806.6	−13.7	−1.7	76	1684.8	1672.3	−12.4	−0.7
32	861.9	811.5	−50.4	−5.8	77	3027.9	3003.6	−24.3	−0.8
33	877.2	853.3	−23.9	−2.7	78	3082.9	3031.2	−51.7	−1.7
34	885.9	879.5	−6.5	−0.7	79	3097.0	3063.6	−33.4	−1.1
35	886.9	907.8	+20.9	+2.4	80	3101.1	3074.8	−26.3	−0.8
36	911.3	916.9	+5.5	+0.6	81	3110.1	3087.2	−22.9	−0.7
37	933.8	932.0	−1.8	−0.2	82	3156.4	3141.2	−15.2	−0.5
38	971.9	944.8	−27.1	−2.8	83	3173.3	3143.8	−29.5	−0.9
39	1002.1	960.8	−41.2	−4.1	84	3195.1	3163.9	−31.2	−1.0
40	1019.4	973.2	−46.2	−4.5	85	3203.7	3175.8	−28.0	−0.9
41	1027.7	1005.2	−22.4	−2.2	86	3213.4	3183.6	−29.8	−0.9
42	1029.4	1015.8	−13.6	−1.3	87	3215.5	3197.5	−18.0	−0.6
43	1032.4	1024.9	−7.4	−0.7	88	3222.2	3204.3	−17.9	−0.6
44	1044.2	1041.6	−2.6	−0.3	89	3230.2	3233.3	+3.1	+0.1
45	1064.2	1046.5	−17.8	−1.7	90	3323.6	3256.4	−67.3	−2.0

```yaml
geom:
 type: redund
 fn: path/to/xyz

calc:
 type: aimnet
 model: 'aimnet2nse_240827_0.jpt'
 charge: 0
 mult: 1  # Singlet (use 2 for doublet radical, 3 for triplet, etc.)

# Option 1: Standard optimizer with analytical Hessian
opt:
 thresh: gau               # Gaussian convergence criteria (tighter than default)
 max_cycles: 500
 hessian_recalc: 10        # Recalculate Hessian every 10 steps
 max_micro_cycles: 100     # Maximum micro-iterations per macro cycle
 hessian_init: calc        # Use analytical Hessian from AIMNet2-NSE
 hessian_update: bfgs      # BFGS update between recalculations
 trust_max: 0.01           # Maximum trust radius (adjust as needed)
 trust_min: 0.001          # Minimum trust radius

# Option 2: L-BFGS optimizer (faster for minima, uses approximate Hessian)
#opt:
# type: lbfgs
# thresh: gau
# max_cycles: 500
```

Figure S17. Representative Pysisyphus YAML configuration file for geometry optimization using AIMNet2-NSE analytical Hessians (requires aimnet2pysis plugin: https://github.com/isayevlab/aimnetcentral)

```
geom:
 type: dlc  # Delocalized internal coordinates
 fn: [path/to/reactant.xyz, path/to/product.xyz]

preopt:  # Preoptimize endpoints before starting GS
 thresh: gau  # Add convergence criteria
 max_cycles: 50     # Cycles for endpoint optimization

cos:
 type: gs           # Growing String method
 max_nodes: 25      # Maximum images in the string
 climb: True        # Enable climbing image for better TS

opt:
 thresh: gau
 max_cycles: 500
 type: string
 align: False

tsopt:
 type: rsirfo       # Restricted-step Image-function RFO
 do_hess: True      # Calculate Hessian at end for frequencies
 thresh: gau
 hessian_recalc: 10 # Recalculate Hessian every 10 steps
 hessian_init: calc # Use analytical Hessian from AIMNet2-NSE
 max_cycles: 500
 trust_max: 0.01
 trust_min: 0.001

irc:
 type: eulerpc      # Euler predictor-corrector
 rms_grad_thresh: 0.0005

endopt:
 thresh: gau        # Add convergence criteria
 do_hess: True      # Calculate frequencies at endpoints

calc:
 type: aimnet
 model: 'aimnet2nse_240827_0.jpt'
 charge: 0
 mult: 1
```

Figure S18. Complete reaction pathway characterization workflow using AIMNet2-NSE: Geometry optimization → Growing String → TS optimization → IRC → endpoint optimization (requires aimnet2pysis plugin: https://github.com/isayevlab/aimnetcentral)

Table S12. Energies for the singlet and triplet 2D scan of AIBN decomposition with the dissociation of N_2 as calculated by ωB97M-D3(BJ)/def2-TZVPP and AIMNet2-NSE on B3LYP-D3(BJ)/def2-TZVP geometries. 2D scan was performed in four equal parts and each part considered 81 geometries in both spin states. The AIMNet2-NSE predictions across the two surfaces are shown in Figure 6. All energy values are reported in atomic units.

C-N$_1$ Distance (Å)	C-N$_2$ Distance (Å)	Singlet		Triplet	
		ωB97M-D3(BJ)	AIMNet2-NSE	ωB97M-D3(BJ)	AIMNet2-NSE
1.4	1.4	-531.372	-531.373	-531.303	-531.3
1.4	1.5625	-531.374	-531.375	-531.303	-531.3
1.4	1.725	-531.362	-531.361	-531.297	-531.297
1.4	1.8875	-531.347	-531.342	-531.291	-531.284
1.4	2.05	-531.331	-531.321	-531.293	-531.283
1.4	2.2125	-531.318	-531.303	-531.298	-531.288
1.4	2.375	-531.306	-531.286	-531.304	-531.294
1.4	2.5375	-531.296	-531.272	-531.308	-531.299
1.4	2.7	-531.288	-531.259	-531.31	-531.302
1.5625	1.4	-531.374	-531.375	-531.303	-531.3

1.5625	1.5625	-531.377	-531.377	-531.309	-531.31
1.5625	1.725	-531.366	-531.365	-531.301	-531.3
1.5625	1.8875	-531.351	-531.347	-531.296	-531.289
1.5625	2.05	-531.337	-531.33	-531.299	-531.291
1.5625	2.2125	-531.324	-531.31	-531.306	-531.297
1.5625	2.375	-531.312	-531.296	-531.312	-531.303
1.5625	2.5375	-531.303	-531.281	-531.316	-531.308
1.5625	2.7	-531.294	-531.27	-531.319	-531.312
1.725	1.4	-531.362	-531.361	-531.297	-531.297
1.725	1.5625	-531.366	-531.365	-531.301	-531.3
1.725	1.725	-531.357	-531.354	-531.294	-531.289
1.725	1.8875	-531.343	-531.337	-531.292	-531.281
1.725	2.05	-531.33	-531.322	-531.298	-531.288
1.725	2.2125	-531.317	-531.308	-531.306	-531.296
1.725	2.375	-531.307	-531.292	-531.312	-531.303
1.725	2.5375	-531.298	-531.279	-531.317	-531.308
1.725	2.7	-531.29	-531.268	-531.32	-531.312
1.8875	1.4	-531.347	-531.342	-531.291	-531.284
1.8875	1.5625	-531.352	-531.347	-531.296	-531.289
1.8875	1.725	-531.343	-531.337	-531.292	-531.281
1.8875	1.8875	-531.33	-531.32	-531.293	-531.281
1.8875	2.05	-531.319	-531.306	-531.302	-531.294
1.8875	2.2125	-531.308	-531.296	-531.311	-531.302
1.8875	2.375	-531.3	-531.282	-531.318	-531.311
1.8875	2.5375	-531.292	-531.272	-531.323	-531.317
1.8875	2.7	-531.285	-531.264	-531.326	-531.32
2.05	1.4	-531.331	-531.321	-531.293	-531.283
2.05	1.5625	-531.337	-531.33	-531.299	-531.291
2.05	1.725	-531.33	-531.322	-531.298	-531.289
2.05	1.8875	-531.319	-531.306	-531.302	-531.294
2.05	2.05	-531.31	-531.295	-531.312	-531.306
2.05	2.2125	-531.301	-531.287	-531.322	-531.315
2.05	2.375	-531.294	-531.278	-531.33	-531.324
2.05	2.5375	-531.288	-531.269	-531.335	-531.33
2.05	2.7	-531.283	-531.263	-531.338	-531.333
2.2125	1.4	-531.318	-531.303	-531.298	-531.288
2.2125	1.5625	-531.324	-531.312	-531.306	-531.297
2.2125	1.725	-531.317	-531.308	-531.306	-531.296
2.2125	1.8875	-531.308	-531.296	-531.311	-531.302
2.2125	2.05	-531.301	-531.287	-531.322	-531.315
2.2125	2.2125	-531.295	-531.28	-531.333	-531.324
2.2125	2.375	-531.289	-531.273	-531.341	-531.333
2.2125	2.5375	-531.284	-531.266	-531.346	-531.338
2.2125	2.7	-531.281	-531.26	-531.349	-531.342
2.375	1.4	-531.306	-531.286	-531.304	-531.294
2.375	1.5625	-531.313	-531.296	-531.312	-531.303
2.375	1.725	-531.307	-531.292	-531.312	-531.303
2.375	1.8875	-531.3	-531.281	-531.318	-531.31
2.375	2.05	-531.294	-531.276	-531.33	-531.324
2.375	2.2125	-531.29	-531.27	-531.341	-531.333
2.375	2.375	-531.286	-531.263	-531.349	-531.342
2.375	2.5375	-531.283	-531.26	-531.354	-531.348
2.375	2.7	-531.28	-531.257	-531.357	-531.351
2.5375	1.4	-531.296	-531.272	-531.308	-531.299

2.5375	1.5625	-531.303	-531.282	-531.316	-531.308
2.5375	1.725	-531.298	-531.279	-531.317	-531.308
2.5375	1.8875	-531.292	-531.272	-531.323	-531.317
2.5375	2.05	-531.288	-531.268	-531.335	-531.33
2.5375	2.2125	-531.285	-531.263	-531.346	-531.339
2.5375	2.375	-531.283	-531.26	-531.354	-531.348
2.5375	2.5375	-531.28	-531.258	-531.36	-531.354
2.5375	2.7	-531.278	-531.257	-531.363	-531.357
2.7	1.4	-531.288	-531.259	-531.31	-531.302
2.7	1.5625	-531.294	-531.27	-531.319	-531.312
2.7	1.725	-531.29	-531.269	-531.32	-531.312
2.7	1.8875	-531.285	-531.264	-531.326	-531.32
2.7	2.05	-531.283	-531.263	-531.338	-531.333
2.7	2.2125	-531.281	-531.26	-531.349	-531.342
2.7	2.375	-531.28	-531.258	-531.358	-531.352
2.7	2.5375	-531.278	-531.257	-531.363	-531.358
2.7	2.7	-531.276	-531.256	-531.366	-531.361
2.7	1.4	-531.287	-531.258	-531.31	-531.302
2.7	1.5625	-531.293	-531.269	-531.319	-531.312
2.7	1.725	-531.289	-531.268	-531.32	-531.312
2.7	1.8875	-531.284	-531.263	-531.326	-531.32
2.7	2.05	-531.282	-531.263	-531.338	-531.333
2.7	2.2125	-531.281	-531.26	-531.349	-531.342
2.7	2.375	-531.28	-531.257	-531.358	-531.352
2.7	2.5375	-531.278	-531.257	-531.363	-531.358
2.7	2.7	-531.276	-531.256	-531.366	-531.361
2.85	1.4	-531.28	-531.25	-531.311	-531.304
2.85	1.5625	-531.287	-531.261	-531.32	-531.314
2.85	1.725	-531.283	-531.261	-531.321	-531.314
2.85	1.8875	-531.28	-531.259	-531.328	-531.322
2.85	2.05	-531.278	-531.26	-531.34	-531.335
2.85	2.2125	-531.278	-531.259	-531.351	-531.344
2.85	2.375	-531.277	-531.257	-531.359	-531.354
2.85	2.5375	-531.276	-531.256	-531.365	-531.36
2.85	2.7	-531.396	-531.388	-531.368	-531.363
3	1.4	-531.275	-531.247	-531.312	-531.306
3	1.5625	-531.281	-531.258	-531.321	-531.315
3	1.725	-531.278	-531.256	-531.322	-531.315
3	1.8875	-531.274	-531.255	-531.329	-531.323
3	2.05	-531.274	-531.26	-531.341	-531.336
3	2.2125	-531.375	-531.36	-531.354	-531.346
3	2.375	-531.386	-531.375	-531.362	-531.356
3	2.5375	-531.393	-531.383	-531.367	-531.363
3	2.7	-531.397	-531.388	-531.37	-531.366
3.15	1.4	-531.315	-531.313	-531.313	-531.307
3.15	1.5625	-531.323	-531.321	-531.321	-531.316
3.15	1.725	-531.318	-531.315	-531.323	-531.316
3.15	1.8875	-531.31	-531.302	-531.329	-531.323
3.15	2.05	-531.303	-531.287	-531.343	-531.337
3.15	2.2125	-531.378	-531.362	-531.354	-531.347
3.15	2.375	-531.388	-531.376	-531.362	-531.357
3.15	2.5375	-531.395	-531.385	-531.367	-531.363
3.15	2.7	-531.399	-531.389	-531.371	-531.367
3.3	1.4	-531.316	-531.314	-531.313	-531.308

3.3	1.5625	-531.323	-531.322	-531.322	-531.317
3.3	1.725	-531.318	-531.316	-531.323	-531.317
3.3	1.8875	-531.311	-531.303	-531.329	-531.323
3.3	2.05	-531.304	-531.288	-531.344	-531.337
3.3	2.2125	-531.378	-531.362	-531.355	-531.347
3.3	2.375	-531.389	-531.377	-531.363	-531.357
3.3	2.5375	-531.395	-531.385	-531.368	-531.363
3.3	2.7	-531.399	-531.39	-531.371	-531.367
3.45	1.4	-531.316	-531.314	-531.314	-531.308
3.45	1.5625	-531.324	-531.322	-531.322	-531.317
3.45	1.725	-531.319	-531.317	-531.323	-531.317
3.45	1.8875	-531.311	-531.304	-531.33	-531.323
3.45	2.05	-531.304	-531.289	-531.344	-531.337
3.45	2.2125	-531.373	-531.364	-531.355	-531.347
3.45	2.375	-531.389	-531.377	-531.363	-531.357
3.45	2.5375	-531.396	-531.385	-531.368	-531.363
3.45	2.7	-531.4	-531.39	-531.371	-531.367
3.6	1.4	-531.316	-531.314	-531.314	-531.309
3.6	1.5625	-531.324	-531.322	-531.322	-531.317
3.6	1.725	-531.319	-531.317	-531.323	-531.318
3.6	1.8875	-531.311	-531.305	-531.332	-531.324
3.6	2.05	-531.304	-531.29	-531.345	-531.338
3.6	2.2125	-531.373	-531.364	-531.356	-531.348
3.6	2.375	-531.389	-531.378	-531.364	-531.358
3.6	2.5375	-531.396	-531.386	-531.369	-531.364
3.6	2.7	-531.4	-531.39	-531.372	-531.368
3.75	1.4	-531.315	-531.314	-531.314	-531.309
3.75	1.5625	-531.324	-531.323	-531.322	-531.318
3.75	1.725	-531.319	-531.318	-531.324	-531.318
3.75	1.8875	-531.311	-531.305	-531.333	-531.325
3.75	2.05	-531.304	-531.29	-531.345	-531.339
3.75	2.2125	-531.373	-531.365	-531.356	-531.349
3.75	2.375	-531.389	-531.378	-531.364	-531.359
3.75	2.5375	-531.396	-531.386	-531.369	-531.365
3.75	2.7	-531.4	-531.39	-531.373	-531.368
3.9	1.4	-531.315	-531.314	-531.314	-531.309
3.9	1.5625	-531.324	-531.323	-531.323	-531.318
3.9	1.725	-531.319	-531.318	-531.324	-531.319
3.9	1.8875	-531.311	-531.305	-531.333	-531.325
3.9	2.05	-531.304	-531.29	-531.345	-531.339
3.9	2.2125	-531.378	-531.363	-531.356	-531.349
3.9	2.375	-531.389	-531.378	-531.364	-531.359
3.9	2.5375	-531.394	-531.386	-531.37	-531.365
3.9	2.7	-531.401	-531.396	-531.373	-531.368
1.4	2.7	-531.287	-531.258	-531.31	-531.302
1.4	2.85	-531.28	-531.25	-531.311	-531.304
1.4	3	-531.275	-531.247	-531.312	-531.306
1.4	3.15	-531.315	-531.313	-531.313	-531.307
1.4	3.3	-531.316	-531.314	-531.313	-531.308
1.4	3.45	-531.316	-531.314	-531.314	-531.308
1.4	3.6	-531.316	-531.314	-531.314	-531.309
1.4	3.75	-531.315	-531.314	-531.314	-531.309
1.4	3.9	-531.315	-531.314	-531.314	-531.309
1.5625	2.7	-531.293	-531.269	-531.319	-531.312

1.5625	2.85	-531.287	-531.262	-531.32	-531.314
1.5625	3	-531.282	-531.259	-531.321	-531.315
1.5625	3.15	-531.279	-531.258	-531.321	-531.316
1.5625	3.3	-531.324	-531.322	-531.322	-531.317
1.5625	3.45	-531.324	-531.322	-531.322	-531.317
1.5625	3.6	-531.324	-531.323	-531.322	-531.317
1.5625	3.75	-531.324	-531.323	-531.322	-531.318
1.5625	3.9	-531.324	-531.323	-531.323	-531.318
1.725	2.7	-531.289	-531.268	-531.32	-531.312
1.725	2.85	-531.284	-531.262	-531.321	-531.314
1.725	3	-531.279	-531.258	-531.322	-531.315
1.725	3.15	-531.274	-531.255	-531.323	-531.316
1.725	3.3	-531.271	-531.252	-531.323	-531.317
1.725	3.45	-531.269	-531.25	-531.323	-531.317
1.725	3.6	-531.268	-531.25	-531.323	-531.318
1.725	3.75	-531.319	-531.318	-531.324	-531.318
1.725	3.9	-531.319	-531.318	-531.324	-531.319
1.8875	2.7	-531.284	-531.263	-531.326	-531.32
1.8875	2.85	-531.28	-531.259	-531.328	-531.322
1.8875	3	-531.275	-531.257	-531.329	-531.323
1.8875	3.15	-531.271	-531.254	-531.329	-531.324
1.8875	3.3	-531.268	-531.251	-531.329	-531.324
1.8875	3.45	-531.266	-531.249	-531.332	-531.322
1.8875	3.6	-531.264	-531.246	-531.332	-531.324
1.8875	3.75	-531.268	-531.25	-531.333	-531.324
1.8875	3.9	-531.268	-531.249	-531.333	-531.325
2.05	2.7	-531.283	-531.263	-531.338	-531.333
2.05	2.85	-531.278	-531.261	-531.34	-531.335
2.05	3	-531.275	-531.26	-531.341	-531.336
2.05	3.15	-531.271	-531.26	-531.341	-531.337
2.05	3.3	-531.363	-531.339	-531.344	-531.337
2.05	3.45	-531.364	-531.339	-531.344	-531.337
2.05	3.6	-531.364	-531.339	-531.345	-531.338
2.05	3.75	-531.364	-531.339	-531.345	-531.339
2.05	3.9	-531.363	-531.339	-531.345	-531.339
2.2125	2.7	-531.281	-531.26	-531.349	-531.342
2.2125	2.85	-531.278	-531.259	-531.351	-531.344
2.2125	3	-531.275	-531.26	-531.352	-531.345
2.2125	3.15	-531.376	-531.36	-531.352	-531.346
2.2125	3.3	-531.376	-531.361	-531.355	-531.347
2.2125	3.45	-531.377	-531.361	-531.355	-531.347
2.2125	3.6	-531.377	-531.361	-531.356	-531.348
2.2125	3.75	-531.377	-531.361	-531.356	-531.349
2.2125	3.9	-531.377	-531.361	-531.356	-531.349
2.375	2.7	-531.28	-531.257	-531.357	-531.351
2.375	2.85	-531.277	-531.257	-531.359	-531.353
2.375	3	-531.387	-531.376	-531.36	-531.354
2.375	3.15	-531.388	-531.377	-531.361	-531.356
2.375	3.3	-531.389	-531.376	-531.363	-531.357
2.375	3.45	-531.389	-531.377	-531.363	-531.357
2.375	3.6	-531.389	-531.377	-531.364	-531.358
2.375	3.75	-531.389	-531.378	-531.364	-531.359
2.375	3.9	-531.389	-531.378	-531.364	-531.359
2.5375	2.7	-531.278	-531.257	-531.363	-531.357

2.5375	2.85	-531.276	-531.256	-531.365	-531.359
2.5375	3	-531.393	-531.384	-531.366	-531.36
2.5375	3.15	-531.394	-531.384	-531.367	-531.363
2.5375	3.3	-531.394	-531.385	-531.367	-531.364
2.5375	3.45	-531.394	-531.385	-531.368	-531.364
2.5375	3.6	-531.394	-531.385	-531.367	-531.364
2.5375	3.75	-531.394	-531.386	-531.367	-531.364
2.5375	3.9	-531.394	-531.386	-531.367	-531.363
2.7	2.7	-531.276	-531.256	-531.366	-531.361
2.7	2.85	-531.274	-531.256	-531.368	-531.362
2.7	3	-531.273	-531.257	-531.369	-531.364
2.7	3.15	-531.397	-531.389	-531.371	-531.367
2.7	3.3	-531.397	-531.39	-531.371	-531.367
2.7	3.45	-531.401	-531.395	-531.371	-531.367
2.7	3.6	-531.401	-531.396	-531.371	-531.367
2.7	3.75	-531.401	-531.396	-531.371	-531.367
2.7	3.9	-531.401	-531.396	-531.371	-531.367
2.7	2.7	-531.276	-531.256	-531.368	-531.363
2.7	2.85	-531.274	-531.255	-531.369	-531.365
2.7	3	-531.398	-531.389	-531.37	-531.366
2.7	3.15	-531.399	-531.39	-531.371	-531.367
2.7	3.3	-531.399	-531.39	-531.371	-531.367
2.7	3.45	-531.4	-531.389	-531.371	-531.367
2.7	3.6	-531.4	-531.39	-531.371	-531.367
2.7	3.75	-531.4	-531.39	-531.371	-531.367
2.7	3.9	-531.4	-531.39	-531.371	-531.367
2.85	2.7	-531.274	-531.255	-531.369	-531.365
2.85	2.85	-531.273	-531.255	-531.371	-531.366
2.85	3	-531.271	-531.256	-531.372	-531.367
2.85	3.15	-531.399	-531.392	-531.372	-531.368
2.85	3.3	-531.4	-531.392	-531.372	-531.369
2.85	3.45	-531.4	-531.392	-531.372	-531.369
2.85	3.6	-531.4	-531.393	-531.372	-531.369
2.85	3.75	-531.4	-531.393	-531.372	-531.368
2.85	3.9	-531.4	-531.393	-531.372	-531.368
3	2.7	-531.398	-531.389	-531.37	-531.366
3	2.85	-531.4	-531.391	-531.372	-531.367
3	3	-531.401	-531.392	-531.373	-531.368
3	3.15	-531.402	-531.392	-531.373	-531.369
3	3.3	-531.402	-531.393	-531.373	-531.37
3	3.45	-531.402	-531.394	-531.373	-531.37
3	3.6	-531.402	-531.394	-531.373	-531.37
3	3.75	-531.402	-531.395	-531.373	-531.369
3	3.9	-531.402	-531.396	-531.373	-531.369
3.15	2.7	-531.399	-531.39	-531.371	-531.367
3.15	2.85	-531.401	-531.392	-531.372	-531.368
3.15	3	-531.402	-531.392	-531.373	-531.369
3.15	3.15	-531.403	-531.393	-531.373	-531.37
3.15	3.3	-531.403	-531.394	-531.374	-531.37
3.15	3.45	-531.403	-531.395	-531.374	-531.371
3.15	3.6	-531.403	-531.395	-531.373	-531.37
3.15	3.75	-531.403	-531.396	-531.373	-531.37
3.15	3.9	-531.403	-531.397	-531.373	-531.37
3.3	2.7	-531.399	-531.39	-531.371	-531.367

3.3	2.85	-531.401	-531.391	-531.372	-531.369
3.3	3	-531.403	-531.392	-531.373	-531.37
3.3	3.15	-531.403	-531.394	-531.374	-531.371
3.3	3.3	-531.404	-531.395	-531.374	-531.371
3.3	3.45	-531.404	-531.395	-531.374	-531.371
3.3	3.6	-531.404	-531.396	-531.373	-531.371
3.3	3.75	-531.404	-531.397	-531.373	-531.371
3.3	3.9	-531.404	-531.397	-531.373	-531.37
3.45	2.7	-531.4	-531.389	-531.371	-531.367
3.45	2.85	-531.402	-531.391	-531.372	-531.369
3.45	3	-531.403	-531.392	-531.373	-531.37
3.45	3.15	-531.404	-531.394	-531.373	-531.37
3.45	3.3	-531.404	-531.395	-531.374	-531.371
3.45	3.45	-531.404	-531.396	-531.374	-531.371
3.45	3.6	-531.404	-531.396	-531.373	-531.371
3.45	3.75	-531.404	-531.397	-531.373	-531.37
3.45	3.9	-531.404	-531.397	-531.373	-531.37
3.6	2.7	-531.4	-531.39	-531.371	-531.367
3.6	2.85	-531.402	-531.392	-531.372	-531.369
3.6	3	-531.403	-531.393	-531.373	-531.37
3.6	3.15	-531.404	-531.394	-531.373	-531.37
3.6	3.3	-531.404	-531.395	-531.373	-531.371
3.6	3.45	-531.404	-531.396	-531.373	-531.371
3.6	3.6	-531.404	-531.397	-531.373	-531.371
3.6	3.75	-531.404	-531.397	-531.373	-531.37
3.6	3.9	-531.404	-531.398	-531.373	-531.37
3.75	2.7	-531.4	-531.39	-531.371	-531.367
3.75	2.85	-531.402	-531.392	-531.372	-531.368
3.75	3	-531.403	-531.393	-531.373	-531.369
3.75	3.15	-531.404	-531.395	-531.373	-531.37
3.75	3.3	-531.404	-531.396	-531.373	-531.371
3.75	3.45	-531.404	-531.397	-531.373	-531.37
3.75	3.6	-531.404	-531.397	-531.373	-531.37
3.75	3.75	-531.404	-531.398	-531.373	-531.37
3.75	3.9	-531.404	-531.398	-531.373	-531.37
3.9	2.7	-531.4	-531.391	-531.371	-531.367
3.9	2.85	-531.402	-531.393	-531.372	-531.368
3.9	3	-531.403	-531.394	-531.373	-531.369
3.9	3.15	-531.404	-531.395	-531.373	-531.37
3.9	3.3	-531.404	-531.396	-531.373	-531.37
3.9	3.45	-531.404	-531.397	-531.373	-531.37
3.9	3.6	-531.404	-531.398	-531.373	-531.37
3.9	3.75	-531.404	-531.398	-531.373	-531.37
3.9	3.9	-531.404	-531.398	-531.373	-531.37

References

[1] L. Pattanaik, A. Menon, V. Settels, K. A. Spiekermann, Z. Tan, F. H. Vermeire, F. Sandfort, P. Eiden, W. H. Green, ConfSolv: Prediction of Solute Conformer-Free Energies across a Range of Solvents, *J. Phys. Chem. B* **2023**, *127*, 10151–10170.
[2] P. M. Zimmerman, Growing string method with interpolation and optimization in internal coordinates: Method and examples, *J. Chem. Phys.* **2013**, *138*, 184102.
[3] K. A. Spiekermann, X. Dong, A. Menon, W. H. Green, M. Pfeifle, F. Sandfort, O. Welz, M. Bergeler, Accurately Predicting Barrier Heights for Radical Reactions in Solution Using Deep Graph Networks, *J. Phys. Chem. A* **2024**, *128*, 8384–8403.
[4] C. Bannwarth, S. Ehlert, S. Grimme, GFN2-xTB—An Accurate and Broadly Parametrized Self-Consistent Tight-Binding Quantum Chemical Method with Multipole Electrostatics and Density-Dependent Dispersion Contributions, *J. Chem. Theory Comput.* **2019**, *15*, 1652–1671.
[5] C. Bannwarth, E. Caldeweyher, S. Ehlert, A. Hansen, P. Pracht, J. Seibert, S. Spicher, S. Grimme, Extended tight-binding quantum chemistry methods, *WIREs Comput. Mol. Sci.* **2021**, *11*, e1493.
[6] G. Henkelman, B. P. Uberuaga, H. Jónsson, A climbing image nudged elastic band method for finding saddle points and minimum energy paths, *J. Chem. Phys.* **2000**, *113*, 9901–9904.
[7] R. Ahlrichs, M. Bär, M. Häser, H. Horn, C. Kölmel, Electronic structure calculations on workstation computers: The program system turbomole, *Chem. Phys. Lett.* **1989**, *162*, 165–169.
[8] S. G. Balasubramani, G. P. Chen, S. Coriani, M. Diedenhofen, M. S. Frank, Y. J. Franzke, F. Furche, R. Grotjahn, M. E. Harding, C. Hättig, A. Hellweg, B. Helmich-Paris, C. Holzer, U. Huniar, M. Kaupp, A. Marefat Khah, S. Karbalaei Khani, T. Müller, F. Mack, B. D. Nguyen, S. M. Parker, E. Perlt, D. Rappoport, K. Reiter, S. Roy, M. Rückert, G. Schmitz, M. Sierka, E. Tapavicza, D. P. Tew, C. van Wüllen, V. K. Voora, F. Weigend, A. Wodyński, J. M. Yu, TURBOMOLE: Modular program suite for ab initio quantum-chemical and condensed-matter simulations, *J. Chem. Phys.* **2020**, *152*, 184107.
[9] A. D. Becke, Density-functional thermochemistry. III. The role of exact exchange, *J. Chem. Phys.* **1993**, *98*, 5648–5652.
[10] C. Lee, W. Yang, R. G. Parr, Development of the Colle-Salvetti correlation-energy formula into a functional of the electron density, *Phys. Rev. B* **1988**, *37*, 785–789.
[11] S. Grimme, J. Antony, S. Ehrlich, H. Krieg, A consistent and accurate ab initio parametrization of density functional dispersion correction (DFT-D) for the 94 elements H-Pu, *J. Chem. Phys.* **2010**, *132*, 154104.
[12] S. Grimme, S. Ehrlich, L. Goerigk, Effect of the damping function in dispersion corrected density functional theory, *J. Comput. Chem.* **2011**, *32*, 1456–1465.
[13] J. Tao, J. P. Perdew, V. N. Staroverov, G. E. Scuseria, Climbing the Density Functional Ladder: Nonempirical Meta--Generalized Gradient Approximation Designed for Molecules and Solids, *Phys. Rev. Lett.* **2003**, *91*, 146401.
[14] A. Klamt, G. Schüürmann, COSMO: a new approach to dielectric screening in solvents with explicit expressions for the screening energy and its gradient, *J. Chem. Soc., Perkin Trans. 2* **1993**, 799–805.
[15] M. Panić, V. Gunjević, K. Radošević, M. Cvjetko Bubalo, K. K. Ganić, I. R. Redovniković, COSMOtherm as an Effective Tool for Selection of Deep Eutectic Solvents Based Ready-To-Use Extracts from Graševina Grape Pomace, *Molecules* **2021**, *26*, 4722.
[16] M. J. Frisch, G. W. Trucks, H. B. Schlegel, G. E. Scuseria, M. A. Robb, J. R. Cheeseman, G. Scalmani, V. Barone, G. A. Petersson, H. Nakatsuji, X. Li, M. Caricato, A. V. Marenich, J. Bloino, B. G. Janesko, R. Gomperts, B. Mennucci, H. P. Hratchian, J. V. Ortiz, A. F. Izmaylov, J. L. Sonnenberg, Williams, F. Ding, F. Lipparini, F. Egidi, J. Goings, B. Peng, A. Petrone, T. Henderson, D. Ranasinghe, V. G. Zakrzewski, J. Gao, N. Rega, G. Zheng, W. Liang, M. Hada, M. Ehara, K. Toyota, R. Fukuda, J. Hasegawa, M. Ishida, T. Nakajima, Y. Honda, O. Kitao, H. Nakai, T. Vreven, K. Throssell, J. A. Montgomery Jr., J. E. Peralta, F. Ogliaro, M. J. Bearpark, J. J. Heyd, E. N. Brothers, K. N. Kudin, V. N. Staroverov, T. A. Keith, R. Kobayashi, J. Normand, K. Raghavachari, A. P. Rendell, J. C. Burant, S. S. Iyengar, J. Tomasi, M. Cossi, J. M. Millam, M. Klene, C. Adamo, R. Cammi, J. W. Ochterski, R. L. Martin, K. Morokuma, O. Farkas, J. B. Foresman, D. J. Fox, Gaussian 16 Rev. C.01, **2016**.
[17] B. Peters, A. Heyden, A. T. Bell, A. Chakraborty, A growing string method for determining transition states: Comparison to the nudged elastic band and string methods, *J. Chem. Phys.* **2004**, *120*, 7877–7886.
[18] P. Pracht, S. Grimme, C. Bannwarth, F. Bohle, S. Ehlert, G. Feldmann, J. Gorges, M. Müller, T. Neudecker, C. Plett, S. Spicher, P. Steinbach, P. A. Wesołowski, F. Zeller, CREST—A program for the exploration of low-energy molecular chemical space, *J. Chem. Phys.* **2024**, *160*, 114110.
[19] N. Mardirossian, M. Head-Gordon, ωB97M-V: A combinatorially optimized, range-separated hybrid, meta-GGA density functional with VV10 nonlocal correlation, *J. Chem. Phys.* **2016**, *144*, 214110.
[20] J.-D. Chai, M. Head-Gordon, Long-range corrected hybrid density functionals with damped atom–atom dispersion corrections, *Phys. Chem. Chem. Phys.* **2008**, *10*, 6615–6620.

[21] S. Grimme, C. Bannwarth, P. Shushkov, A Robust and Accurate Tight-Binding Quantum Chemical Method for Structures, Vibrational Frequencies, and Noncovalent Interactions of Large Molecular Systems Parametrized for All spd-Block Elements (Z = 1-86), *J. Chem. Theory Comput.* **2017**, *13*, 1989–2009.
[22] F. Neese, Software update: The ORCA program system—Version 5.0, *WIREs Comput. Mol. Sci.* **2022**, *12*, e1606.
[23] M. D. Hanwell, D. E. Curtis, D. C. Lonie, T. Vandermeersch, E. Zurek, G. R. Hutchison, Avogadro: an advanced semantic chemical editor, visualization, and analysis platform, *J. Cheminform.* **2012**, *4*, 17.
[24] E. C. Meng, T. D. Goddard, E. F. Pettersen, G. S. Couch, Z. J. Pearson, J. H. Morris, T. E. Ferrin, UCSF ChimeraX: Tools for structure building and analysis, *Protein Sci.* **2023**, *32*, e4792.
[25] L. Liu, H. Jiang, P. He, W. Chen, X. Liu, J. Gao, J. Han, On the Variance of the Adaptive Learning Rate and Beyond, **2021**, arXiv preprint, DOI: 10.48550/arXiv.1908.03265.
[26] J. Steinmetzer, S. Kupfer, S. Gräfe, pysisyphus: Exploring potential energy surfaces in ground and excited states, *Int. J. Quantum Chem.* **2021**, *121*, e26390.
[27] R. Fletcher, *Practical Methods of Optimization*, John Wiley & Sons, **2013**.
[28] A. Banerjee, N. Adams, J. Simons, R. Shepard, Search for stationary points on surfaces, *J. Phys. Chem.* **1985**, *89*, 52–57.
[29] E. Besalú, J. M. Bofill, On the automatic restricted-step rational-function-optimization method, *Theor. Chem. Acc.* **1998**, *100*, 265–274.
[30] H. P. Hratchian, M. J. Frisch, H. B. Schlegel, Steepest descent reaction path integration using a first-order predictor–corrector method, *J. Chem. Phys.* **2010**, *133*, 224101.
[31] L. Goerigk, A. Hansen, C. Bauer, S. Ehrlich, A. Najibi, S. Grimme, A look at the density functional theory zoo with the advanced GMTKN55 database for general main group thermochemistry, kinetics and noncovalent interactions, *Phys. Chem. Chem. Phys.* **2017**, *19*, 32184–32215.
[32] J. Řezáč, Non-Covalent Interactions Atlas benchmark data sets 5: London dispersion in an extended chemical space, *Phys. Chem. Chem. Phys.* **2022**, *24*, 14780–14793.
